# Single-cell RNA sequencing reveals cellular diversity and gene expression dynamics in maize root development

**DOI:** 10.3389/fpls.2025.1666531

**Published:** 2025-11-27

**Authors:** Jianwen Bian, Zelong Zhuang, Rui Tang, Wanling Ta, Zhenping Ren, Yunling Peng

**Affiliations:** 1 College of Agronomy, Gansu Agricultural University, Lanzhou, China; 2 State Key Laboratory of Aridland Crop Science, Gansu Agricultural University, Lanzhou, China; 3 Gansu Key Laboratory of Crop Improvement & Germplasm Enhancement, Gansu Agricultural University, Lanzhou, China

**Keywords:** scRNA-seq, maize root development, cell heterogeneity, pseudotime, STP4

## Abstract

**Introduction:**

Roots are essential for plant growth, functioning in nutrient and water uptake and anchorage.

**Methods:**

To elucidate the molecular basis of maize root development at single-cell resolution, single-cell RNA sequencing (scRNA-seq) was performed on maize root tips.

**Results:**

This analysis identified nine cell types and ten transcriptionally distinct clusters based on marker and cluster-specific gene expression. Cyclin gene profiling revealed M-phase enrichment across most root tissues, indicating active cell division in the meristem. Further investigation uncovered cell-type expression patterns of hormone-related genes in maize roots, which diverged from those observed in A. thaliana and rice. Pseudotime analysis reconstructed the developmental trajectory from early to mature cortex, revealing candidate regulators of cell fate determination. Weighted gene co-expression network analysis (WGCNA) identified Zm00001d021775 (sugar transport protein STP4) as a hub gene in the mature cortex.

**Discussion:**

Functional inference suggests STP4 promotes early seedling growth by facilitating glucose transport into glycolysis and the TCA cycle. These findings provide a high-resolution map of transcriptional landscapes in maize roots, offering new insights into cellular heterogeneity, developmental regulation, and potential molecular targets for enhancing root function and crop resilience.

## Introduction

Root system is an essential organ for plant growth and development, responsible for the absorption of nutrients and water from the soil, as well as providing stability to the plant (Meister et al., 2014). Plant root systems are mainly categorized into two types: the taproot systems found in dicots and the fibrous root systems characteristic of monocots (Liu et al., 2021). In most dicots, such as *A. thaliana*, the root system consists of a single primary root and numerous lateral roots derived from it. Conversely, monocots predominantly rely on a complex fibrous root system composed of multiple post-embryonic adventitious roots that arise from the base of the stem and extend downward (Cheng et al., 2023). It is noteworthy that current research on the regulatory mechanisms of root system development largely focuses on dicot models, particularly *A. thaliana*. However, since most global staple crops are monocots, a significant gap exists in our systematic understanding of the transcriptional regulatory programs specific to cell types within their root systems. Therefore, conducting an in-depth analysis of root system development in monocot crops would address this knowledge gap and provide theoretical support for improving crop stress resistance.

scRNA-seq is an innovative high-throughput sequencing technology capable of amplifying and sequencing mRNA within individual cells, thereby accurately assessing gene expression levels in each cell (Tanay and Regev, 2017). This technology enables the revelation of the expression status of all genes across the entire genome at single-cell resolution, aiding in uncovering cellular heterogeneity and deepening our understanding of cellular fate determination and developmental genetic mechanisms (Giacomello, 2021). In recent years, scRNA-seq has been successfully applied to various plant species, significantly advancing our knowledge of plant tissue architecture and developmental dynamics (Iqbal et al., 2020). For instance, Zhang and Denyer et al. revealed the heterogeneity of *A. thaliana* root tip cells at the single-cell level, depicted a comprehensive developmental landscape of *A. thaliana*, and reconstructed the developmental trajectory of root apical meristem cells (Denyer et al., 2019; Zhang et al., 2019). Zhang et al. elucidated the single-cell heterogeneity in rice roots, constructed differentiation trajectories of rice epidermal cells and ground tissue cells, clarified the correlation between gene expression and chromatin accessibility during root apical stem cell differentiation, and comparatively analyzed evolutionary conservation of root tip cell types between the monocot rice and the dicot *A. thaliana* (Zhang et al., 2021). Wang et al. mapped the single-cell transcriptomic landscape of rice, compared homologous tissue gene expression in leaves and roots, and explored cell-type-specific transcriptional regulation and proportional changes under abiotic stress, offering new insights into the transcriptional regulatory mechanisms during rice development and stress responses (Wang et al., 2021). Moreover, scRNA-seq has been extensively utilized in studies on plants such as wheat (Zhang et al., 2023), cotton (Li et al., 2024), tomato (Tian et al., 2020), peanuts (Liu et al., 2021), tea (Wang et al., 2022), tobacco (Jin et al., 2024), and soybean (Cervantes-Perez et al., 2024). These studies confirm a high degree of heterogeneity in plant tissues and identify specific marker genes associated with cell division and cell differentiation. Nevertheless, research on the mechanisms of root system development at the single-cell level in crops remains relatively limited, lacking systematic and thorough exploration.

Maize (*Zea mays* L.), a globally significant monocot cereal crop, plays a decisive role in plant growth, yield formation, and environmental adaptation through its root system architecture. Under unfavorable soil conditions, optimizing root system architecture to enhance water and nutrient absorption capacity is crucial for developing high-yielding, stress-resistant maize varieties (Rogers and Benfey, 2015; Ren et al., 2022). With the advancement of scRNA-seq technology, its application in maize has become increasingly widespread, providing new perspectives for dissecting cellular heterogeneity and functional regulatory mechanisms. For example, Li et al. employed scRNA-seq to reveal the heterogeneity of maize root cells and analyzed the response mechanisms of different cell types to nitrate stress (Li et al., 2022). Wang et al. constructed a single-cell transcriptomic atlas of maize roots under heat stress, discovering that the cortex is the principal root cell type responding to heat stress with the highest number of differentially expressed genes, whose developmental trajectory is preferentially affected under heat stress (Wang et al., 2025). Additionally, Cao et al. revealed cell type-specific transcriptional regulatory networks regulating fungal invasion in maize roots through single-cell RNA sequencing profiling (Cao et al., 2023). In addition to the root system, scRNA-seq has also been applied to study other important maize tissues, including female inflorescences (Xu et al., 2021), mesophyll (Tao et al., 2022), and shoot apices (Ma et al., 2025), further expanding our understanding of maize multi-tissue developmental processes and functional differentiation.

In this study, a single-cell transcriptomic atlas of maize root tips from 7-day-old plants was constructed using scRNA-seq technology, and major cell types was identified. Through analyzing differentially expressed genes (DEGs) and cyclin genes in various cell clusters, the biological functions of different cell types during root system development were revealed. Furthermore, through comparison of the spatial expression patterns of plant hormone-related genes, differences among maize, *Arabidopsis*, and rice were uncovered. Pseudotime trajectory analysis was employed to reconstruct the developmental path from early-to-mature stages of maize root cortex cells. Based on this, weighted gene co-expression network analysis was used to construct gene co-expression networks in different cell types, leading to the identification of a candidate gene closely associated with cortex development. This gene encodes a sugar transporter and may play a critical role in energy supply and morphogenesis during early maize root development. Collectively, this study provides a profound insight into the transcriptional regulatory characteristics of maize root system development at the single-cell level, laying a theoretical foundation for genetic improvement of maize root systems.

## Materials and methods

### Plant material and growth conditions

Seedlings of the maize inbred line B73 were used for root scRNA-seq experiments. The seeds were sterilized using a 10% sodium hypochlorite (NaClO) solution for ten minutes, followed by three washes with distilled water. After sterilization, the seeds were placed on germination papers, which were then rolled and positioned vertically within a sealed plastic bag. The paper roll was incubated at a temperature regime of 25 °C during the light period and 20 °C during the dark period, with a 10-hour light and 14-hour dark cycle for 7 days. On the 7 days of culture, the root tips of the primary roots were excised for subsequent experiments (**Supplementary Figure S1**).

### Protoplast isolation of root tips for scRNA-seq

Root tip regions (~2 cm in length from the root tip) were harvested and cut into ~ 0.5 mm segments. Tissues were digested in enzyme solution containing 1.5% (w/v) cellulase R-10, 0.15% (w/v) macerozyme, 0.5% (w/v) hemicellulase, 20 mM KCl, 10 mM CaCl_2_, 0.1% (w/v) BSA, 20 mM MES, and 0.6 M mannitol (pH 5.7). The mixture was vacuum-infiltrated for 10 min and then incubated in the dark at 25 °C with gentle shaking (85 rpm) for 2 h. After digestion, the suspension was filtered through a 40 μm nylon mesh and centrifuged at 250 × g for 3 min. The pellet was washed twice with pre-chilled PBS containing 0.6 M mannitol. Protoplasts were resuspended in PBS supplemented with 0.6 M mannitol. Cell concentration and viability were assessed using 0.4% Trypan Blue staining. Only samples with viability >85% were used for sequencing. Protoplasts were adjusted to a final concentration of 1000–2000 cells/μL in ice-cold PBS with 0.6 M mannitol and immediately loaded onto the 10x Genomics Chromium platform.

### scRNA-seq library construction and sequencing

The scRNA-seq library construction and sequencing were completed by Gene Denovo Biotechnology (Guangzhou, China). In brief, cellular suspensions were loaded onto a 10x Genomics Chromium^TM^ System using the Chromium Next GEM Single Cell 3’ Reagent Kit (v3.1, 10x Genomics) according to the manufacturer’s protocol. Gel beads were co-encapsulated with single cells and partitioning oil to generate Gel Bead-In-Emulsions (GEMs). Upon dissolution of the gel bead in a GEM, reverse transcription primers containing (i) an Illumina® R1 sequence (read1 sequencing primer), (ii) a 16 bp 10x Barcode, (iii) a 12bp Unique Molecular Identifier (UMI), and (iv) a 30-bp poly-dT sequence were released into each GEM. Reverse transcription was performed within the GEMs to generate barcoded, full-length cDNAs from polyadenylated mRNA. After GEM breakage, cDNAs were purified using SPRI (Solid Phase Reversible Immobilization) magnetic beads (AMPure XP). Full-length cDNAs were amplified by PCR to generate sufficient copies for library construction. The cDNA product was then fragmented, end-repaired, and size-selected. sequencing connectors (P5 and P7) and sequencing primers (R1 and R2) were added through a second round of PCR to generate the final sequencing library. Libraries were sequenced using the PE150 sequencing mode on the Illumina HiSeq 4000 sequencing platform.

### Pre-processing of raw scRNA-seq data

Raw sequencing data was processed using Cellranger (https://support.10xgenomics.com/single-cell-gene-expression/software/overview/welcome), where the low-quality reads were filtered. Through alignment with the B73 reference genome (Zm-B73-REFERENCE-GRAMENE-4.0) and annotation of reads to specific genes, an unfiltered feature-barcode matrix was generated after UMI correction and counting. Cellranger then utilized this matrix to distinguish between cells and non-cells, producing a rank-plot graph for visual representation of cell identification results. Utilizing the UMI barcodes, gene quantification was performed based on the corrected UMIs and valid cell identification outcomes. Cells with the criteria of gene count between 390 and 13000 per cell, unique molecular identifier (UMI) counts less than 47000 per cell, and percentage of mitochondrial genes less than 10% were filtered using Seurat (v2.0.4) R package (Butler et al., 2018). After filtering, the data were normalized using the 'LogNormalize' method, and highly variable genes were identified for downstream analysis. The expression values were scaled, and potential confounding factors such as mitochondrial percentage were regressed out during the scaling process.

### Cell clustering, visualization, and cell type annotation

Cell clustering workflow follows established single-cell RNA-seq analysis standards. Seurat implements a graph-based clustering approach. Distances between the cells were calculated based on previously identified principal components (PCs). Briefly, Seurat embeds cells in a shared-nearest neighbor (SNN) graph, with edges drawn between cells *via* similar gene expression patterns. To partition this graph into highly interconnected quasi-cliques or communities, we first constructed the SNN graph based on the Euclidean distance in principal component analysis (PCA) space and refined the edge weights between any two cells based on the shared overlap in their local neighborhoods (Jaccard distance). We then cluster cells using the Louvain method to maximize modularity with a resolution of 0.5 (Rotta and Noack, 2011).

For visualization, two nonlinear dimensionality reduction techniques were applied: t-distributed stochastic neighbor embedding (t-SNE) (Laurens and Hinton, 2008) and uniform manifold approximation and projection (UMAP) (Becht et al., 2019). Both were computed based on the same 20 PCs.

For the annotation of cell types, we first use Single R for cell annotation, which is based on correlating gene expression of reference cell types with single-cell expression. First, a Spearman coefficient is calculated for single-cell expression with each of the samples in the reference data set. Next, multiple correlation coefficients per cell type are aggregated to provide a single value per cell type per single cell. Finally, SingleR reruns the correlation analysis, but only for the top cell types from the previous step. The analysis was performed only on variable genes. The cell type corresponding to the top value after the last run is assigned to the single cell (Aran et al., 2019). In addition, we collected known cell-type-specific marker genes from the literature and the plant cell marker database for cell annotation.

### RNA *in situ* hybridization

RNA *in situ* hybridization was performed as described by Du et al. (2021) with minor modifications (Du et al., 2021). Briefly, maize root tips were fixed in RNase-free FAA (50% ethanol, 5% acetic acid, and 5% formaldehyde) (Coolaber, SL16222) for 24 h at 4 °C. Fixed root tips were dehydrated through a series of ethanol (50%, 60%, 70%, 80%, 95%, and 100%), cleared in histoclear, then embedded in paraplast wax. Tissue sections (8 μm) were cut using a Leica RM2135 microtome (Leica, Germany) and mounted on Probeon Plus Slides. Before hybridization, slides were deparaffinized, rehydrated, treated with proteinase K (20 μg/mL, 37 °C, 15 min), postfixed in 4% paraformaldehyde, acetylated, and prehybridized at 55 °C for 2 h. DIG-labeled RNA probes were synthesized *in vitro* (Roche), denatured, and hybridized overnight at 55 °C. After stringent washes, slides were blocked and incubated with anti-DIG-AP antibody (1:2000, 4 °C, overnight). Signals were detected with NBT/BCIP in AP buffer (dark, 4–6 h), stopped with PBS, and counterstained with 0.02% Fast Red or mounted directly. Images were captured using a Nikon DS-Ri2 DIC microscope. Primer sequences for all genes are listed in **Supplementary Table S1**.

### Differentially expressed genes (up-regulation) analysis

Expression values of each gene in the given cluster were compared against the rest of the cells using the Wilcoxon rank sum test (Camp et al., 2017). Genes were considered significantly up-regulated based on the following criteria: (1) log2 fold-change > 0.36 (equivalent to 1.28-fold overexpressed in the target cluster). (2) genes had to be expressed in more than 25% of the cells belonging to the target cluster. (3) p-value ≤ 0.01 (Benjamini-Hochberg correction for multiple testing).

Functional enrichment analyses of up-regulated genes were conducted using the OmicShare online platform (https://www.omicshare.com/tools/). Gene Ontology (GO) terms and KEGG pathways were analyzed using the hypergeometric test, with significance set at adjusted p-value < 0.05. Only terms containing at least five annotated genes were considered for interpretation. The background gene set was defined as all genes expressed in the dataset.

### Cell cycle analysis

To infer the cell cycle phase of individual cells, we first curated gene sets associated with distinct cell cycle phases based on literature (Macosko et al., 2015) and the CycleBase database (https://cyclebase.org), Based on the gene set for cell cycle assessment, namely G1, S, G2, and M, were constructed respectively according to different cell cycle periods. The cells were classified into five types using the above four gene sets, namely the cell types at each stage of the cycle, and the non-cycling cells that do not express the genes of the cycle characteristic proteins.

The AddModuleScore scoring function of the Seurat software scores the possible cell cycle phases based on the average expression level of the gene set of cell cycle characteristic proteins in the cells. First, sort the genes by their average expression levels from low to high, and randomly divide them into n modules. Randomly select an equal amount of genes from the module where the periodic characteristic protein gene set is located as the background gene set. For a single cell, take the difference between the average expression levels of the periodic characteristic protein gene set and the background gene set in the cell as the cell cycle score. Score each periodic characteristic protein gene set in sequence, and the period with the highest score represents the cell cycle period of the cell, and the corresponding score is the cell cycle score. Pseudo-time analysis.

Pseudo-time trajectory analysis of single-cell transcriptomes was conducted using Monocle 2 (Trapnell et al., 2014). First, Monocle2 uses the DifferentialGeneTest function to identify DEGs based on pseudo-time values. Subsequently, the dimensionality of the data is reduced to two dimensions (max_components = 2, method = 'DDRTree'), while Monocle sorts the units. Next, we use the orderCells function to sort the cells and plot_cell_trajectory to visualize the trajectory in a reduced-dimensional space. In addition, we use orderCells to specify the start again with the root_state parameter. Finally, Monocle uses the branching expression analysis modeling (BEAM) method to analyze cell data after pseudo-time and specified nodes, mining DEG related to branching, and using the plot_generes_branched_heapmap function to visualize genes that depend on branching.

### Weighted gene co-expression network analysis

Co-expression networks were constructed using WGCNA (v1.47) package in R (Langfelder and Horvath, 2008). After filtering genes, gene expression values were imported into WGCNA to construct co-expression modules using the automatic network construction function blockwiseModules with default settings, except that the power is 11, TOMType is unsigned, mergeCutHeight is 0.15, minModuleSize is 50. Intramodular connectivity (K.in) and module correlation degree (MM) of each gene were calculated by R package of WGCNA and genes with high connectivity tended to be hub genes, which might have important functions. The networks were visualized using Cytoscape_3.10.1 (Shannon et al., 2003).

## Results

### scRNA-seq and identification of root tip cell clusters

**Figure 1:**
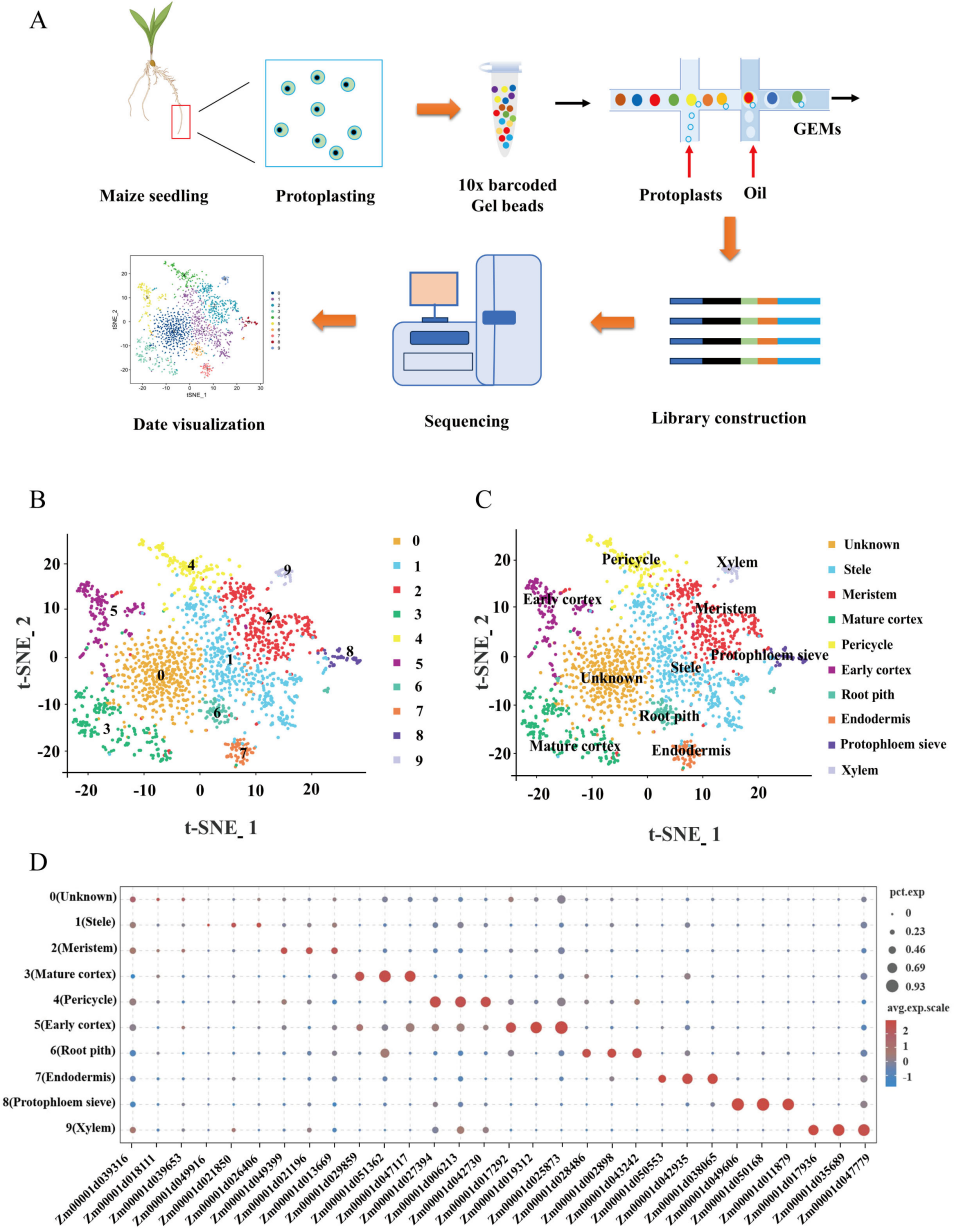
Single-cell RNA-seq and cluster annotation of maize root tips. **(A)** Overview of maize root tips scRNA-seq workflow. **(B, C)** t-SNE visualization for the identification of 10 cell clusters in root tips. Each dot indicates a single cell. Colors in the diagram of the root tip indicate corresponding cell clusters. **(D)** Expression patterns of representative cell-specific genes in 10 cell clusters. Dot diameter indicates the proportion of cluster cells expressing a given gene. Color on the dots indicates the expression level.

To explore the molecular mechanisms underlying maize root development at the single-cell transcriptome level, we utilized maize inbred line B73 root tips from 7-day-old seedlings to generate protoplasts for single-cell transcriptome analysis using the 10× Genomics platform (**Figure 1A**). We captured 1949 high-quality root tip cells (before filter 2700), with a median of 754 unique molecular identifiers (UMIs) per cell (before filter 1040.5), and an average of 595 genes expressed per cell (before filter 762) (**Supplementary Figures S2–S4**). The scRNA-seq data were processed through linear dimensional reduction, followed by visualization using t-SNE and UMAP algorithms. Through unsupervised clustering analysis, we identified 10 distinct cell clusters representing different cell populations within the maize root tip (**Figures 1B, C**; **Supplementary Figure S5**). Furthermore, we characterized cluster-specific gene expression patterns, identifying a set of marker genes that were specifically expressed in one or two clusters (**Figure 1D**).

**Figure 2:**
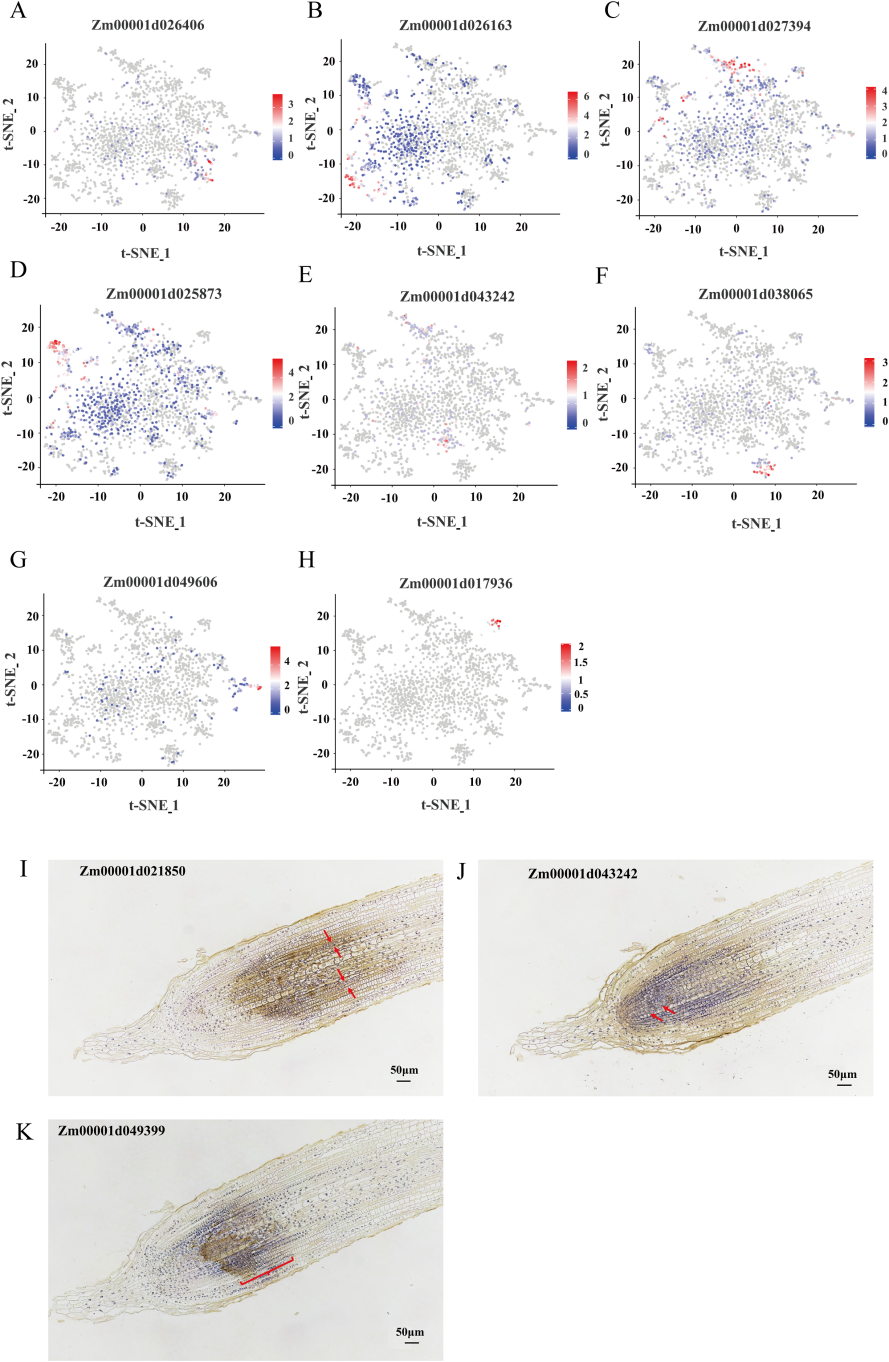
t-SNE visualization of marker genes and RNA *in situ* hybridization validation. **(A-H)** The expression of representative cell type marker genes distributed in t-SNE map. Each dot indicates a single cell. The color of the dots indicates the expression level. **(I-K)** RNA *in situ* hybridization validation of representative cell type marker genes.

To accurately annotate the identified cell clusters, we used well-characterized marker genes previously reported in articles and the single-cell plant databases scPlantDB (He et al., 2024) and PlantscRNAdb (Chen et al., 2021), whose functions and expression patterns have been extensively studied (**Supplementary Table S2**). Specifically, cluster 1 was identified as stele based on the specific expression of marker genes Zm00001d026406 and Zm00001d021850. Cluster 3 was annotated as mature cortex, characterized by the high expression of Zm00001d026163 and Zm00001d029859. Similarly, cluster 4 was designated as pericycle due to the presence of Zm00001d027394 and Zm00001d051478. While cluster 5 was classified as early cortex based on the expression of Zm00001d025873 and Zm00001d017292. Furthermore, cluster 6 was identified as root pith through the expression of Zm00001d043242 and Zm00001d002898, and cluster 7 was annotated as endodermis based on the expression of Zm00001d038065 and Zm00001d042935. Cluster 8 was annotated as protophloem sieve elements, marked by the significant expression of Zm00001d049606 and Zm00001d050168, while cluster 9 was identified as xylem based on the expression of Zm00001d017936 and Zm00001d035689 (**Figures 2A–H**; **Supplementary Figures S6A–H**). Notably, no cluster-specific genes are exclusively expressed in cluster 0, leading to its classification as unknown due to the absence of marker genes for annotation. To validate the accuracy of our cluster annotations, we performed RNA *in situ* hybridization, which confirmed the spatial expression patterns of key marker genes: Zm00001d021850 was specifically localized to the stele, Zm00001d049399 exhibited meristem-specific expression (supporting the annotation of cluster 2 as meristem), and Zm00001d043242 was specifically expressed in the root pith (**Figures 2I–K**). In summary, our single-cell RNA sequencing analysis successfully delineated nine distinct cell types within the maize root tip, highlighting the remarkable cellular heterogeneity of this developmental tissue.

### Differential expressed genes analysis of cell types

**Figure 3:**
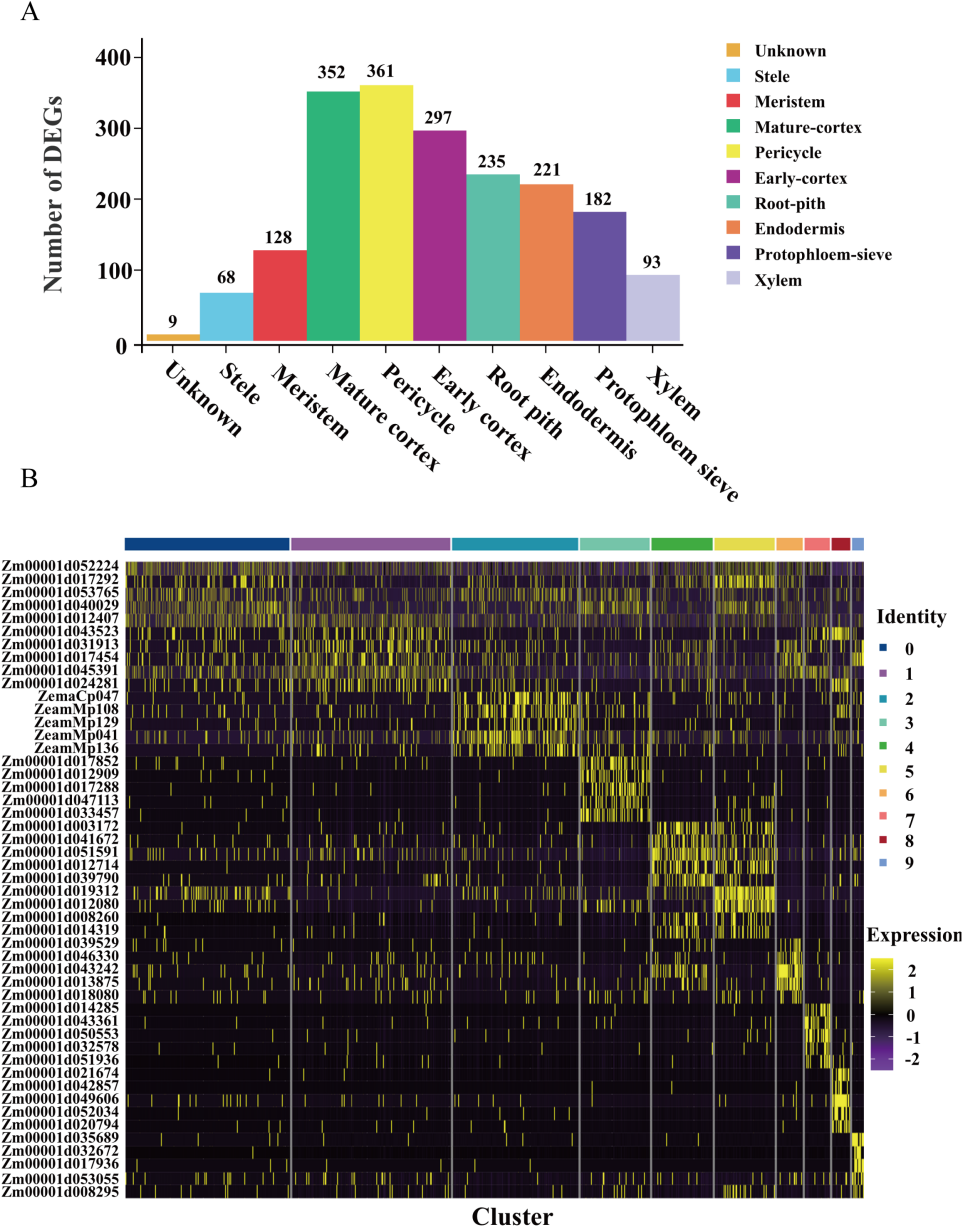
Differential expressed genes analysis of each cell type. **(A)** Statistical histogram of up-regulated genes in different root cell types. **(B)** Heatmap presenting the expression of the top 5 up-regulated genes in 10 cell clusters.

To elucidate the molecular heterogeneity among distinct cell subgroups, we identified upregulated differentially expressed genes (DEGs) across the 10 clusters, resulting in a total of 1946 upregulated DEGs (**Figure 3A**; **Supplementary Table S3**). Notably, the pericycle exhibited the highest number of upregulated DEGs, including Zm00001d003172, Zm00001d041672, Zm00001d051591, Zm00001d012714, and Zm00001d039790, all of which demonstrated significant upregulation within this subgroup (**Figure 3B**). Similarly, mature cortex cells displayed a substantial number of upregulated DEGs, with genes such as Zm00001d017852, Zm00001d012909, Zm00001d017288, Zm00001d047113, and Zm00001d033457 showing pronounced upregulation. In contrast, cluster 0 contained only nine upregulated DEGs and exhibited a dispersed distribution among various cell types on the UMAP plot. This cluster also displayed a lower median gene count (496.5) compared to other subgroups. Further analysis revealed that the nine upregulated genes in cluster 0 were also specifically expressed in other subpopulations, suggesting that cluster 0 likely represents a mixed population of low-quality cells rather than a distinct cell type. Furthermore, differential expression analysis identified genes specifically enriched in distinct cell clusters, which may serve as novel marker genes (**Supplementary Table 2**). Validation through *in situ* hybridization will provide robust experimental evidence to support future cell type annotation.

To explore the biological significance of the DEGs, we performed GO and KEGG pathway enrichment analyses across all clusters. GO analysis revealed significant enrichment in broad biological processes, including organonitrogen compound biosynthetic processes, translation, peptide biosynthetic processes, water transport, peptide metabolic process, and nucleosome assembly (**Supplementary Figure S7**). KEGG pathway analysis further highlighted enrichment in key biological pathways, particularly ribosome biogenesis, metabolic pathways, and protein processing in the endoplasmic reticulum (**Supplementary Figure S8**). These findings collectively underscore the functional diversity and specialized roles of distinct cell subgroups within the maize root tip.

### Cell cycle analysis of cell subgroups

**Figure 4:**
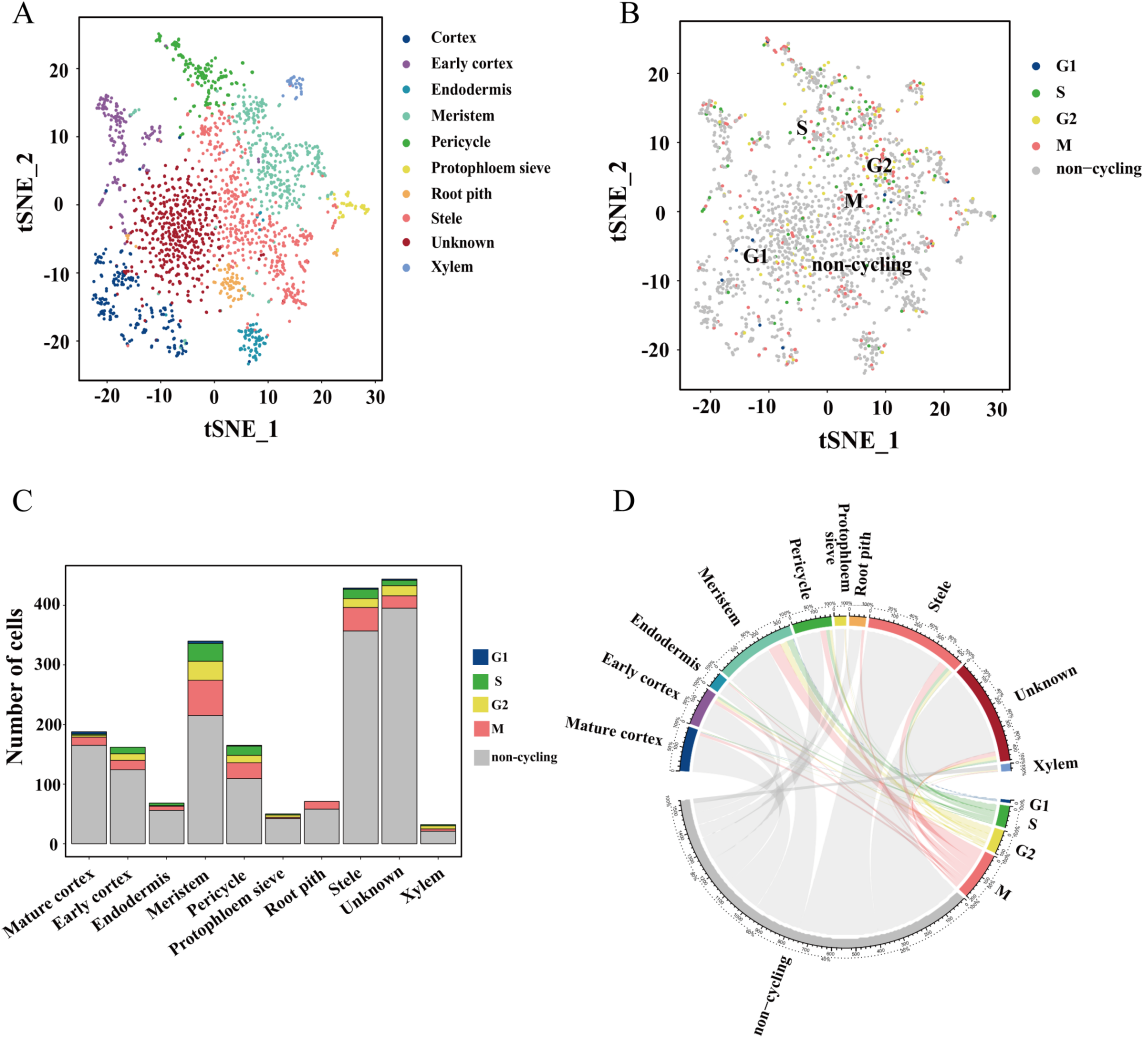
Cell cycle analysis of 10 cell clusters in the maize root tip. **(A, B)** t-SNE visualization of 10 distinct clusters. **(C)** Histogram of the percentage of each cluster. **(D)** Circos diagram of different cell cycle subgroups.

Plant root tip cells exhibit remarkable plasticity, characterized by their dual capacity for multifunctional differentiation and self-proliferation. The proliferation process is tightly regulated by the cell cycle, rendering cell cycle analysis of proliferative cells a critical aspect of understanding root development. We conducted a comprehensive cell cycle analysis across the 10 identified cell subgroups, revealing distinct phase distributions among the clusters (**Figures 4A, B**). Notably, the majority of root tissue cells were found to be non-cycling, with only a subset actively engaged in the cell cycle. Strikingly, the meristem exhibited the highest proportion of cells in the M phase (**Figures 4C, D**), indicating active cell division and underscoring the proliferative nature of this tissue. Phase-specific gene expression analysis identified key regulatory genes associated with distinct cell cycle stages (**Supplementary Table S4**). During the G1 phase, genes such as Zm00001d043330, Zm00001d049333, Zm00001d017252 (PRT1), Zm00001d014971 (MIK1), and Zm00001d002811 (C2C2-GATA-transcription factor 11) were significantly up-regulated. The S phase was marked by the upregulation of Zm00001d051591 (histone 2B5) and Zm00001d053371 (VPS29), while the G2 phase featured elevated expression of ZeamMp044, Zm00001d002546 (H4C7), Zm00001d021433 (HMG-transcription factor 13), COX1, and Zm00001d028183 (CCT3). Furthermore, the M phase was characterized by the upregulation of ZeamMp098 (CCMFN1) and Zm00001d032789 (chaperonin2). These specific genes can serve as marker genes for distinct cell cycle phases (**Supplementary Figure S9A**). A heatmap visualization further confirmed the differential expression patterns of these cyclins across cell cycle phases (**Supplementary Figure S9B**). The precise regulation of these cell cycle genes is essential for orchestrating DNA replication and cell division in a temporally and spatially controlled manner, thereby ensuring the fidelity of genetic information transmission and maintaining cellular integrity. These findings highlight the intricate regulatory mechanisms underlying root tip cell proliferation and provide valuable insights into the molecular basis of plant root development.

### Hormone biosynthesis and response profiles in root tips

**Figure 5:**
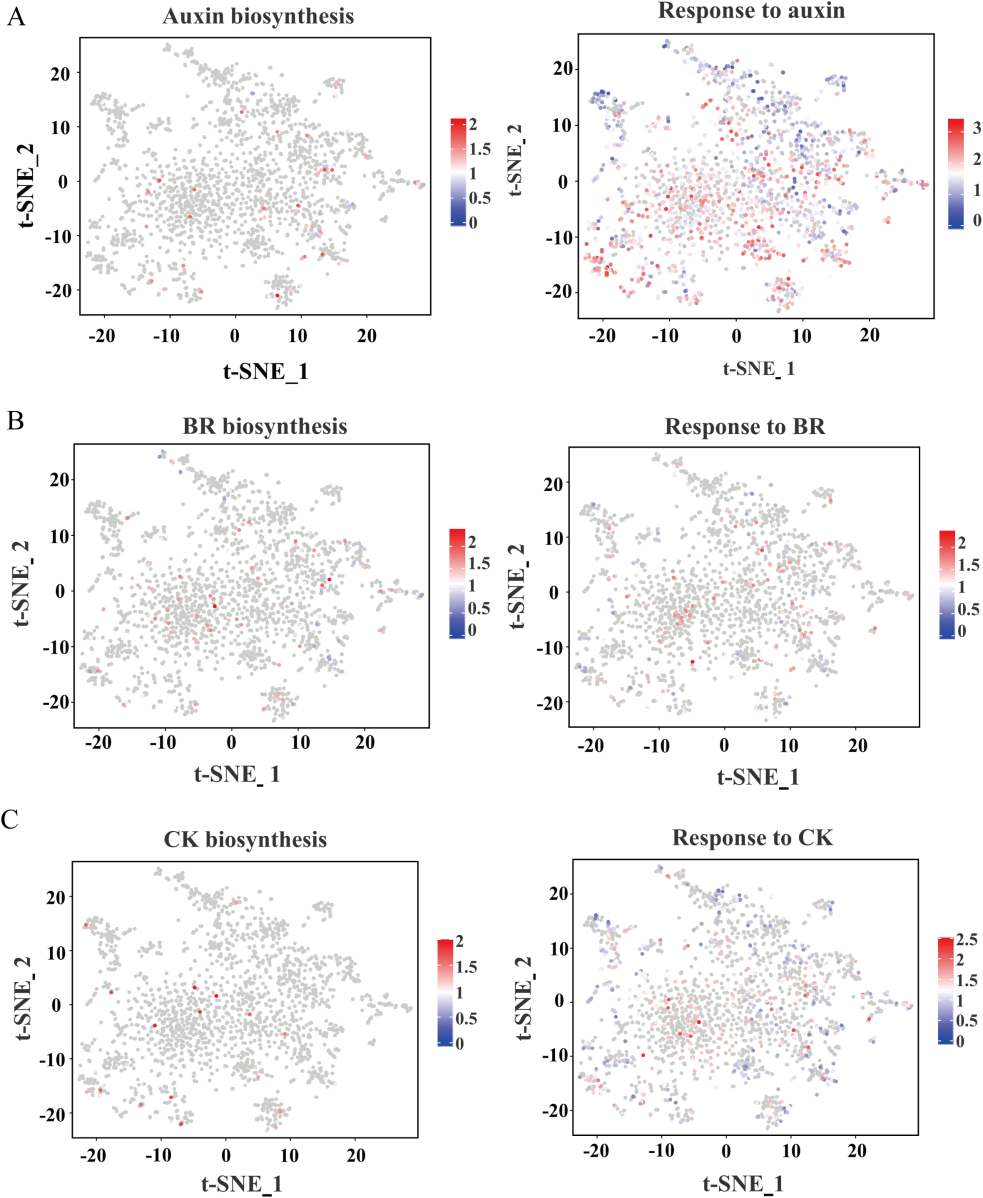
t-SNE visualization of expression patterns related to hormones. **(A)** Auxin biosynthesis and response genes. **(B)** BR biosynthesis and response genes. **(C)** CK biosynthesis and response gene. The colors represent expression levels of these genes in individual cells.

Plant hormones are pivotal regulators of root development and stress responses. To elucidate their spatial expression patterns, we mapped genes associated with hormone biosynthesis and signaling pathways using t-SNE visualization (**Figure 5**). Our analysis indicates that the genes involved in the biosynthesis and response of plant hormones in the root system of maize are expressed in different cell clusters rather than being confined to a single cell type, which is different from that in *Arabidopsis* and rice. For instance, auxin biosynthesis gene *ZmYUC2* and response gene *ZmIAA24* were expressed in distinct cell clusters, with response gene *ZmIAA24* exhibiting higher expression levels (**Figure 5A**), suggesting a prominent role for auxin during this developmental stage. Similarly, brassinosteroid (BR)-related genes, including both biosynthesis and response genes, were predominantly observed in clusters 0 and 1, where they displayed high expression (**Figure 5B**). Cytokinin (CK) biosynthesis genes were notably enriched in clusters 3, 5, and 7, implicating their role in promoting cortex cell division, while CK response genes were more broadly distributed across multiple clusters (**Figure 5C**). A similar spatial expression pattern was observed for other hormones, including gibberellic acid (GA), abscisic acid (ABA), ethylene, and jasmonic acid (JA) (**Supplementary Figure S10**). Notably, the expression levels of ABA biosynthesis-related genes at the root tip are relatively low, which might be the reason why the root tip is in an active developmental state at this time.

### Pseudo-time trajectory analysis of root tip

**Figure 6:**
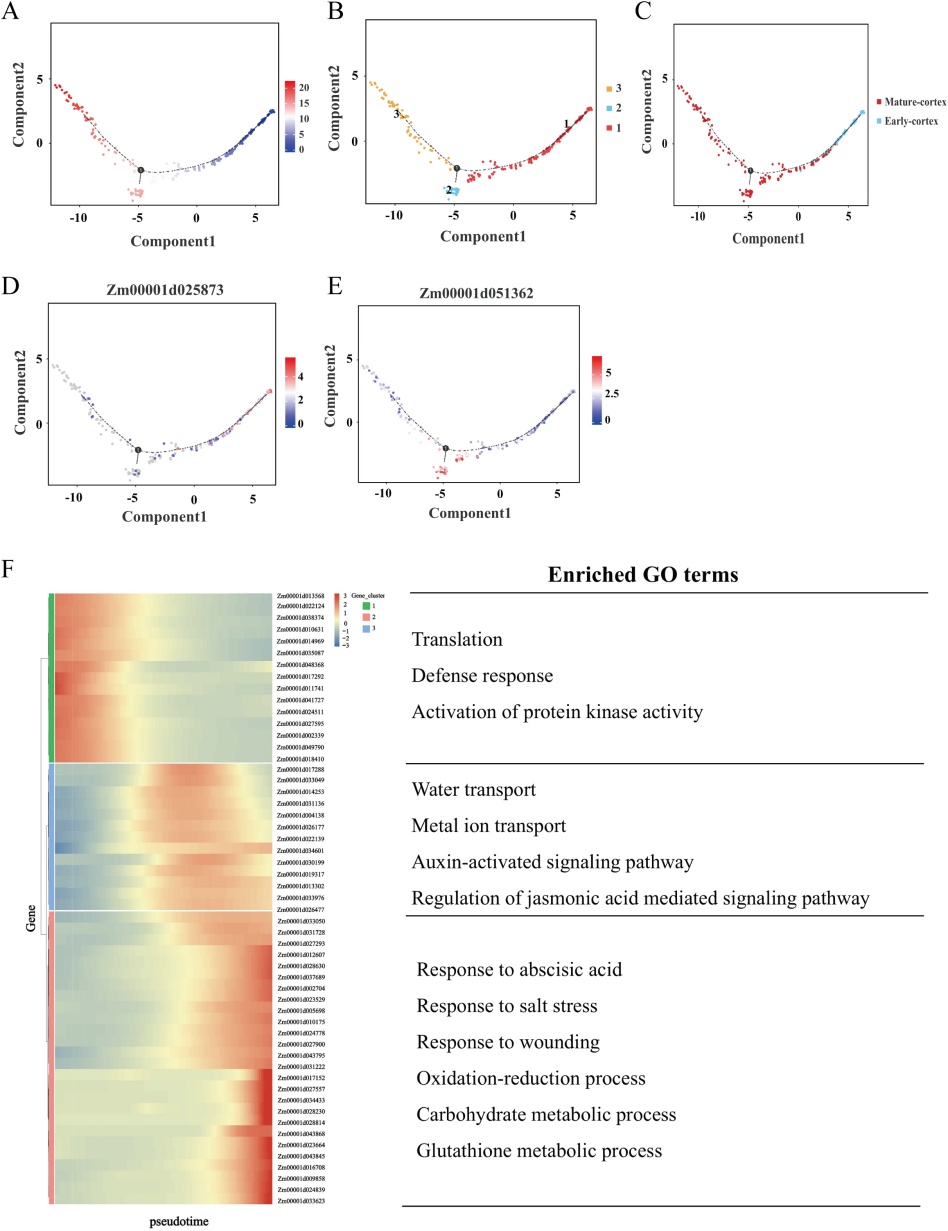
Differentiation trajectory of the cortex in maize root. **(A)** Differentiation trajectory of the early cortex and mature cortex. Each dot indicates a single cell. Different color on the dots indicates the pseudo-time scores. **(B, C)** Cell types labeled on the differentiation trajectory for early cortex and mature cortex. **(D, E)** Cell trajectory analysis of the differentiation fate of early cortex marker gene Zm00001d025873 and mature cortex marker gene Zm00001d051362. Each dot indicates a single cell. Color on the dots indicates the expression abundance in the corresponding parts. **(F)** Heatmap showing the expression of the genes regulating significant enrichment function in two clusters across the pseudo-time. Each row represents one gene. Representative GO terms for each cluster are shown on the right.

Root tip cells undergo a highly dynamic and continuous developmental process during the seedling stage, characterized by progressive differentiation rather than abrupt transitions. scRNA-seq enables to capture of cells in distinct developmental states, facilitating the exploration of the ongoing differentiation pathway in a developmental process. The cortex as a fundamental structural and functional component of the primary root, plays a critical role in root development (Lux et al., 2004). Based on our cell annotations, clusters 5 and 3 were identified as early cortex and mature cortex, respectively. To elucidate the differentiation trajectory of cortex cells, we performed pseudo-temporal analysis to reveal the continuous developmental process. Distinct cell clusters were separately clustered at either end of the pseudo-time axis, and the pseudo-time trajectory represented by color change helped us to pinpoint the beginning of the differentiation process (**Figures 6A, B**). As expected, the analysis confirmed a unidirectional differentiation trajectory from early to mature cortex states (**Figure 6C**). Further examination of the differentiation trajectory revealed stage-specific gene expression patterns: Zm00001d025873, an early cortex marker gene, was highly expressed at the initial branch point, while Zm00001d051362, a mature cortex marker gene, exhibited peak expression at intermediate stages of differentiation (**Figures 6D, E**).

To elucidate the biological mechanisms underlying cortex tissue regulation in maize root tips, we performed DEGs analysis along the pseudo-temporal axis, comparing early and mature cortex states (**Supplementary Table S5**). This analysis identified 666 DEGs, from which 54 representative genes were selected for heatmap visualization. This analysis identified 660 DEGs, from which 54 representative genes were selected for hierarchical clustering and heatmap visualization. These genes segregated into three distinct clusters with unique expression patterns (**Figure 6F**). Genes associated with the early cortex were predominantly enriched in gene cluster 1, while those linked to the mature cortex were primarily distributed across gene clusters 2 and 3. Functional enrichment analysis revealed that gene cluster 1 was significantly associated with biological processes such as translation, defense response, and activation of protein kinase activity. Gene cluster 2 showed enrichment in water transport, metal ion transport, response to oxidative stress, and auxin-activated signaling pathways. Gene cluster 3 was notably enriched in processes including response to abscisic acid, salt stress response, carbohydrate metabolism, and glutathione metabolism. To further investigate the regulatory mechanisms governing cell fate determination, we identified 85 DEGs associated with differentiation fate. Heatmap analysis of these genes revealed five distinct expression clusters, each corresponding to specific differentiation transitions (**Supplementary Figure S11**). For instance, genes within clusters 2 and 3 exhibit relatively high expression levels in state 1, 2 indicates that their role in determining the transition from state 1 to state 2 differentiation fate. Conversely, genes in cluster 4 show elevated expression levels in state 1, 3, suggesting their involvement in determining the transition from state 1 to state 3 differentiation fate.

### Co-expression regulatory network in maize root tips

**Figure 7:**
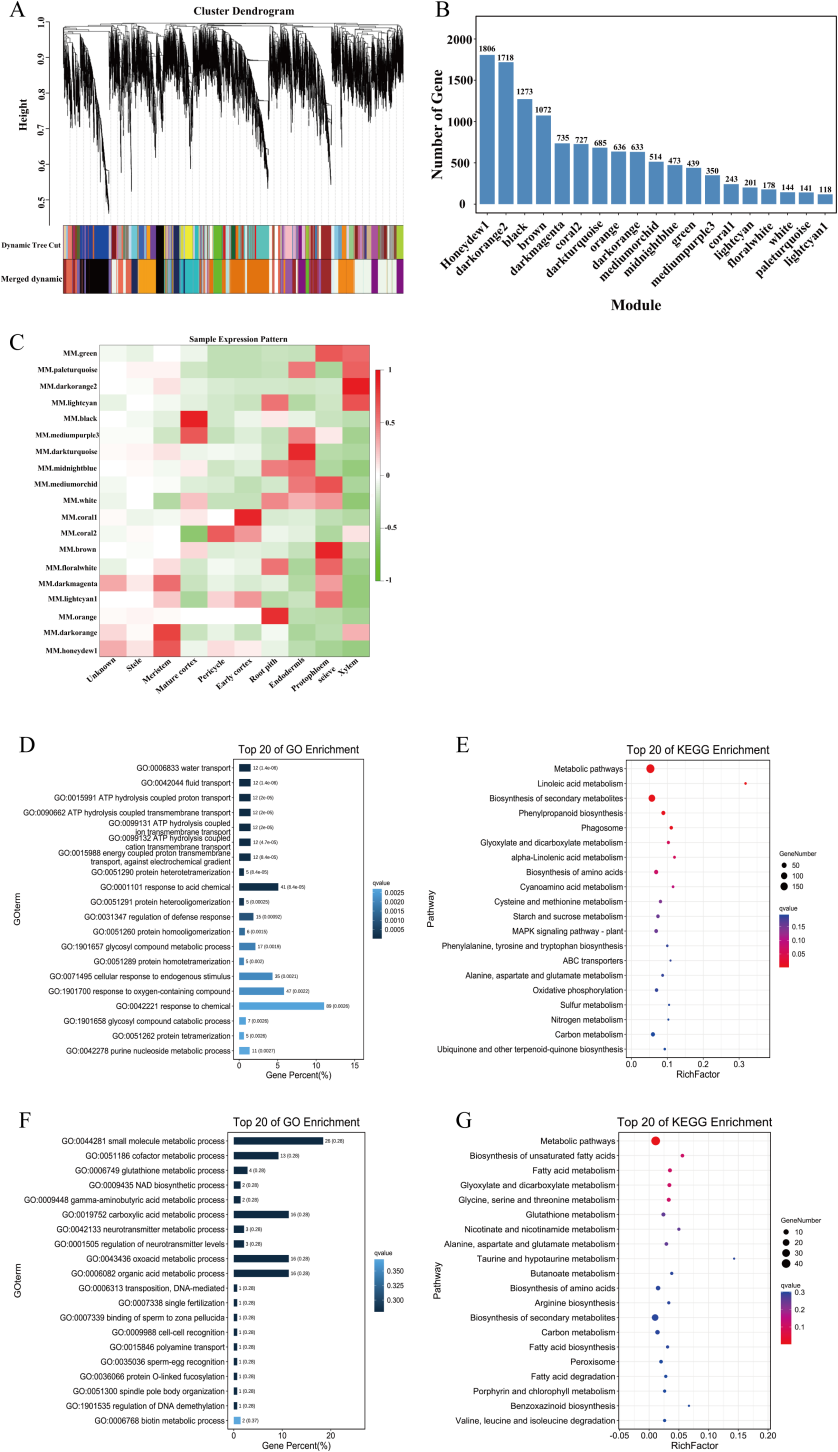
WGCNA of cell clusters in maize root. **(A)** Cluster dendrogram. **(B)** Number of genes of 19 modules. **(C)** Heatmap of sample expression pattern. **(D, E)** The GO and KEGG enrichment of the black module. **(F, G)** The GO and KEGG enrichment of the coral1 module.

To construct a gene co-expression network for each cell cluster, we performed gene co-expression network analysis DEGs in all cell types using WGCNA. The resulting co-expression network comprised 19 modules (**Figure 7A**). Among these, the honeydew1 and darkorange2 modules contained the largest numbers of genes, with 1806 and 1718 genes, respectively, while the lightcyan1 module contained the fewest genes, comprising only 118 genes (**Figure 7B**). Module-cell subset association analysis revealed significant correlations between specific modules and cell subgroups. Notably, the black module exhibited a strong positive correlation with the mature cortex, whereas the coral1 module was significantly associated with the early cortex. In contrast, the stele showed weak correlations with all modules (**Figure 7C**). To further elucidate the specific functions of the black module, we performed GO and KEGG enrichment analyses on the genes within this module. GO analysis revealed that the genes in the black module were significantly enriched in processes related to water transport, liquid transport, ATP hydrolysis-coupled proton transport, and ATP hydrolysis-coupled transmembrane transport (**Figure 7D**). KEGG analysis indicated that the genes in the black module were significantly enriched in metabolic pathways, linolenic acid metabolism, biosynthesis of secondary metabolites, and phenylpropane biosynthesis (**Figure 7E**). Similarly, GO analysis of the coral1 module genes demonstrated significant enrichment in small molecule metabolic process, cofactor metabolic process and glutathione metabolic process (**Figure 7F**). KEGG analysis of the genes in this module showed significant enrichment in metabolic pathways, biosynthesis of unsaturated fatty acids, and fatty acid metabolism (**Figure 7G**).

**Figure 8:**
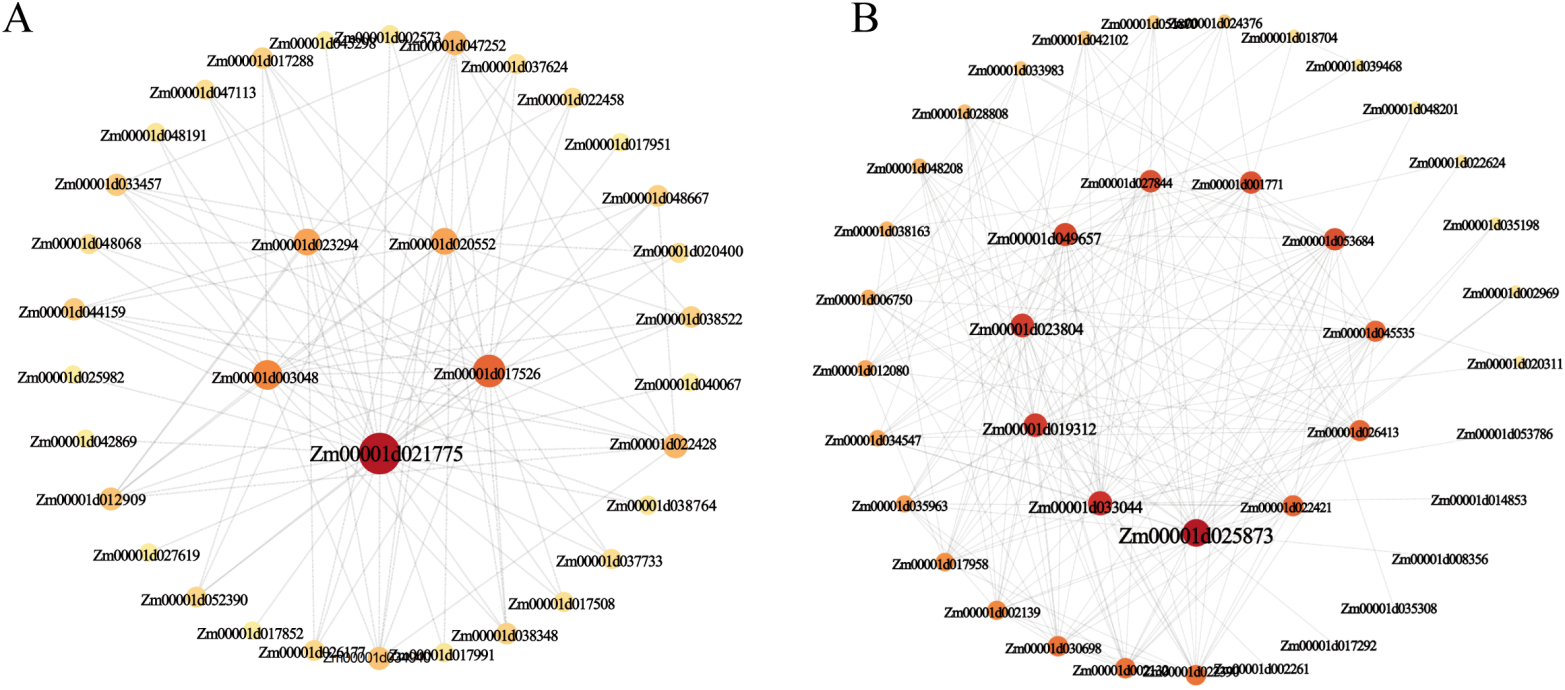
Gene co-expression network of cortex. **(A)** Gene co-expression network and hub genes in two modules of the black module. **(B)** Gene co-expression network and hub genes in two modules of the coral1 module. Nodes of different sizes and colors represent the betweenness centrality value of nodes; larger nodes and darker colors indicate larger values.

To identify hub genes within the target subpopulations, we employed Cytoscape software (v3.10.1) to visualize and analyze the co-expression network of the black module. By selecting the top 200 genes (including duplicates) based on their connectivity weights within the black module, which were subsequently visualized and analyzed through gene connectivity analysis (**Figure 8**). This analysis identified five core hub genes in the black module: Zm00001d021775 (Sugar transport protein 4), Zm00001d017526 (Aquaporin PIP1-2), Zm00001d003048 (Disease resistance protein RPS2), Zm00001d020552 (Senescence-associated protein DH), and Zm00001d023294 (NAC-transcription factor 25) (**Figure 8A**) These findings suggest that these hub genes may play pivotal roles in metabolic regulation and nutrient transport within the mature cortex during root development. Similarly, analysis of the coral1 module revealed core hub genes, including Zm00001d025873 (Defensin-like protein CAL1), Zm00001d033044 (GDSL esterase/lipase), and Zm00001d019312 (Jasmonate-induced protein) (**Figure 8B**). These genes are likely involved in modulating cell wall composition during early cortex development, thereby influencing root elongation and branching, or they may be involved in hormone signaling pathways, which in turn affect root structure development.

## Discussion

### Identification of maize root tip cell types by scRNA-seq

scRNA-seq has revolutionized root transcriptomics by providing unprecedented spatiotemporal resolution, enabling the discovery of cell heterogeneity, marker genes, and developmental trajectories in plant roots (Minne et al., 2022). In *A. thaliana*, scRNA-seq has been extensively applied to uncover these aspects (Denyer et al., 2019; Jean-Baptiste et al., 2019; Zhang et al., 2019). The architecture of maize roots, a monocotyledonous species, differs significantly from that of the dicotyledonous model *A. thaliana*, particularly in terms of root system organization and cell type composition (Meister et al., 2014; Hochholdinger et al., 2018). While root development has been extensively studied in *A. thaliana*, research on other plant species, particularly maize, remains limited. Although scRNA-seq studies on 4-day-old and 7-day-old maize seedlings have identified 9, 11, and 7 distinct cell types, respectively (Li et al., 2022; Cao et al., 2023; Wang et al., 2025), but the full spectrum of cell types in maize root tips has yet to be comprehensively characterized. In our study, we captured 1949 cells of the maize root tip using scRNA-seq and identified 10 major clusters, which were assigned to 9 distinct cell types. Similar to the results of previous studies, and we further identified the early and mature cortex cell types. However, our research failed to capture typical cell types such as root hairs and epidermal cells, which have been reported in recent single-cell studies (Li et al., 2022; Wang et al., 2025). Several technical and biological factors may account for this limitation: First, protoplast preparation bias likely played a significant role. Epidermal cells, particularly root hair cells, possess thickened cell walls and are more fragile during enzymatic digestion. They are therefore prone to lysis or underrepresentation in the final protoplast suspension, leading to their low abundance or complete absence in scRNA-seq libraries (Ren and Wang, 2023). Second, limited sequencing depth and cell number may have reduced the resolution needed to separate transcriptionally similar or rare cell populations (Rizzetto et al., 2017). With 1,949 cells, our dataset provides a valuable snapshot of major cell types but may lack the statistical power to resolve fine subpopulations, especially those with overlapping gene expression profiles, such as non-hair and hair-forming epidermal cells. Lastly, bioinformatic annotation challenges remain, accurate annotation of cell clusters remains a major challenge, primarily due to the limited availability of marker genes and the complexity of cell type identification. Although several databases, such as scPlantDB, PlantscRNAdb, PsctH, and PlantCellMarker, provide valuable resources (Chen et al., 2021; Jin et al., 2022; Xu et al., 2022; Cao et al., 2023), their coverage and specificity for maize root cells are still insufficient. Therefore, future studies should focus on expanding the repertoire of marker genes for maize root development through comprehensive scRNA-seq analyses, integrating multi-omics data, and developing more robust computational tools for cell type annotation. At the same time, the methods for extracting plant protoplasts should be optimized to enable the capture of more protoplast cells.

### Hormonal biosynthesis and response pattern in maize root cell atlas

Phytohormones are crucial regulators of plant development, exerting significant effects on growth even at low concentrations (Garay-Arroyo et al., 2012). In roots, hormone interactions form a complex network that orchestrates development through synergistic or antagonistic effects, ultimately influencing crop performance (Sharma et al., 2021). In recent years, extensive research has been conducted to elucidate these complex regulatory networks. However, it remains unclear how plant roots respond to hormones and whether the synthesis of plant hormones occurs in specific cell types. scRNA-seq has emerged as a powerful tool to unravel the spatiotemporal expression patterns of hormone-related genes in roots. Hu et al. analyzed the cell-type-specific expression of hormone-related genes in tea roots, revealing that exodermal cells specifically accumulated DEGs associated with BRs, ABA, and cytokinins. In the root cap and lateral root cap, auxin- and cytokinin-related genes showed specific expression, likely playing key roles in regulating root architecture. Furthermore, DEGs related to stress-responsive hormones such as ABA and jasmonic acid (JA) were mainly enriched in the endodermis, cortex, and root hairs, suggesting their involvement in environmental sensing and adaptation. Overall, the study highlights a highly cell-type-specific and coordinated hormonal regulatory network in tea roots (Hu et al., 2024). Zhang et al. generated a cellular map of hormone synthesis and response in the root tip of A. thaliana. They found that the sites of hormone synthesis and response exhibit tissue-specific patterns. For example, genes involved in the response to salicylic acid (SA), JA, ABA, and ethylene are specifically expressed in the stele, whereas genes associated with the biosynthesis of indole-3-acetic acid (IAA) and BR are highly enriched in the phloem. These findings provide a foundation for more precise investigations into the regulatory networks governing plant hormone signaling (Zhang et al., 2019). Similar patterns were observed in rice roots by Liu et al. For instance, the genes involved in the synthesis and response of IAA are highly expressed in both the stele cells and the epidermal cells, whereas genes associated with the synthesis and response of other hormones show enrichment in distinct cell types (Liu et al., 2021). Despite these advances, the expression patterns of hormone biosynthesis and response genes in maize roots, which exhibit distinct developmental and architectural features compared to *Arabidopsis* and rice, remain largely unexplored. In this study, we observed that hormone-related genes in maize roots exhibit extensive expression patterns across various cell types, with the differences mainly reflected in expression levels rather than expression locations, suggesting that there may be ecological adaptation differences in root hormone regulation among different plant species (Hochholdinger and Zimmermann, 2008; Singh et al., 2022; Kong et al., 2024). This might reflect a complex regulatory strategy developed by maize, as a large gramineous crop, during its evolution to adapt to its complex root structure and variable field environment. It is worth noting that the expression levels of ABA biosynthesis genes detected in the root tip tissue were relatively low. This might indicate that the root tip is in an active cell division stage, during which the content of ABA is usually low, and it may even be below the detection threshold of single-cell RNA sequencing. Reconstruction of the differentiation trajectory of cortex cells.

Cell developmental trajectories, which describe the transition of cells from one state to another, can be precisely reconstructed using pseudo-time analysis of scRNA-seq (Jean-Baptiste et al., 2019). This approach is essential for understanding fundamental biological processes, including cell growth, differentiation, senescence, and disease progression. Previous studies have successfully applied pseudo-time analysis to reconstruct cell developmental trajectories in roots. For example, Li et al. revealed that root hair cells originate from the differentiation of a subset of epidermal cells, starting from meristematic zone cells (Li et al., 2022). Similarly, Zhang et al. identified two distinct developmental trajectories in *Arabidopsis* roots: one leading to the differentiation of root cap, lateral root cap, epidermis, and root hairs at the distal end, and the other resulting in the differentiation of stele cell types at the proximal end (Zhang et al., 2019). In this study, we reconstructed the developmental trajectory of cortex cells from early to mature stages (**Figure 6**). Analysis of DEGs along the pseudo-time axis revealed distinct functional roles: early cortex development was associated with translation, defense response, and protein kinase activity, while mature cortex development involved water and metal transport, oxidative stress response, and auxin signaling pathways. Furthermore, we analyzed the genes that determine the fate of cell differentiation. For instance, the *TIP1–1* gene is highly expressed in cluster 2. *TIP1–1* is a water channel protein gene and is associated with aging and survival in *Arabidopsis*. The absence of this gene leads to premature plant death (Schussler et al., 2008), suggesting that *TIP1–1* may play a key role in determining the differentiation transition from state 1 to state 2. Concurrently, numerous genes encoding ribosomal proteins (RPs) were found to be highly expressed in cluster 4, indicating their potential involvement in regulating the differentiation transition from state 1 to state 3. RPs are important components of ribosomes. In plants, they not only participate in the basic protein synthesis process, but also have various biological functions such as regulating growth and development and responding to environmental stress (Stępiński, 2025). In *A. thaliana*, *AtRPL14B* plays a unique regulatory role during fertilization and embryonic development. Mutations in the gene encoding *AtRPL14B* disrupt the functions of male and female gametes, leading to reduced pollen grain size and a significant decrease in the competitive ability of pollen tubes (Luo et al., 2020). Similarly, the protein product of *AtRPL18aB* is critically involved in early embryogenesis in *A. thaliana*, particularly in regulating cell division and determining cell fate during the initial stages of embryo development. Mutation of *AtRPL18aB* results in aberrant cell division patterns and ultimately causes arrest of seed development (Yan et al., 2016). These findings indicate that the regulatory functions of ribosomal proteins play a crucial role in key developmental processes during plant growth. Collectively, our findings demonstrate the power of scRNA-seq in reconstructing continuous differentiation trajectories and identifying key genes governing cell fate during root development. However, pseudo-temporal trajectory analysis across different developmental periods remains limited, highlighting the need for more comprehensive studies to elucidate the dynamic regulatory networks underlying cell differentiation.

### Identification of hub gene regulating the development of cortex

The maize root cortex is a key determinant of root morphological diversity and environmental adaptation. It contributes to drought and flood tolerance by regulating water transport and facilitates symbiotic interactions with mycorrhizal fungi, which enhance nutrient uptake and stress resilience (Zhu et al., 2010; Wang et al., 2025). Wang et al. find that cortex size is strongly correlated with heat tolerance, which is experimentally validated using inbred lines and genetic mutation analysis of one candidate gene in maize (Wang et al., 2025). To elucidate the gene regulatory networks in maize root cell types, we performed WGCNA and identified two key modules: the black module was strongly associated with the mature cortex, and the coral1 module correlated with the early cortex. Within the black module, we pinpointed Zm00001d021775 as a hub gene, encoding the sugar transport protein 4 (STP4). *AtSTP1* is a homologue of this gene in *A. thaliana*, which encodes a high-affinity sugar transporter that acts as an H^+^/monosaccharide cotransporter, capable of transporting a wide range of hexoses (Buttner and Sauer, 2000; Cordoba et al., 2015). Otori et al. demonstrated that *AtSTP1* regulates genes involved in shoot branching *via* carbon partitioning in *A. thaliana* (Otori et al., 2019). Monosaccharide transporter (MST) gene family is a complete membrane protein that can participate in the transmembrane transport of monosaccharides, playing critical roles in plant growth, development, and responses to abiotic stresses (Deng et al., 2019; Zhu et al., 2024). STPs, as members of the MST gene family, are highly conserved among plants and mediate hexose transport in cells of different tissues. In rice, Wang et al. demonstrated that *OsMST6* (*OsSTP6*) was a broad-spectrum monosaccharide transporter and its expression was induced by salt stress and sugars (Wang et al., 2008). Luo et al. further revealed that *OsMST6* enhances chilling tolerance *via* ABA signaling, with mutants exhibiting hypersensitivity to cold and overexpression lines displaying resilience (Luo et al., 2024). Additionally, heterologous overexpression of *OsMST6* in *Arabidopsis* improves drought and salt tolerance by reducing water loss and enhancing membrane stability (Monfared et al., 2020). These findings suggest that *MST6* may coordinate sugar and hormone signaling pathways to mitigate abiotic stress. In maize, Zhu et al. conducted a comprehensive genome-wide identification and functional analysis of MST (including STPs) gene family, focusing on their evolutionary relationships and expression patterns under various abiotic stresses and hormone treatments. They found that the ABA response element ABRE exists in most MST member promoter regions, indicating that the expression of MST members might be involved in the ABA signaling pathway (Zhu et al., 2024). Based on these insights, we hypothesize that STP4 may play a similar role in regulating root growth in maize, potentially integrating sugar and hormone signaling to enhance stress tolerance and developmental plasticity.

### Regulatory network of STP4 in maize root tip development

**Figure 9:**
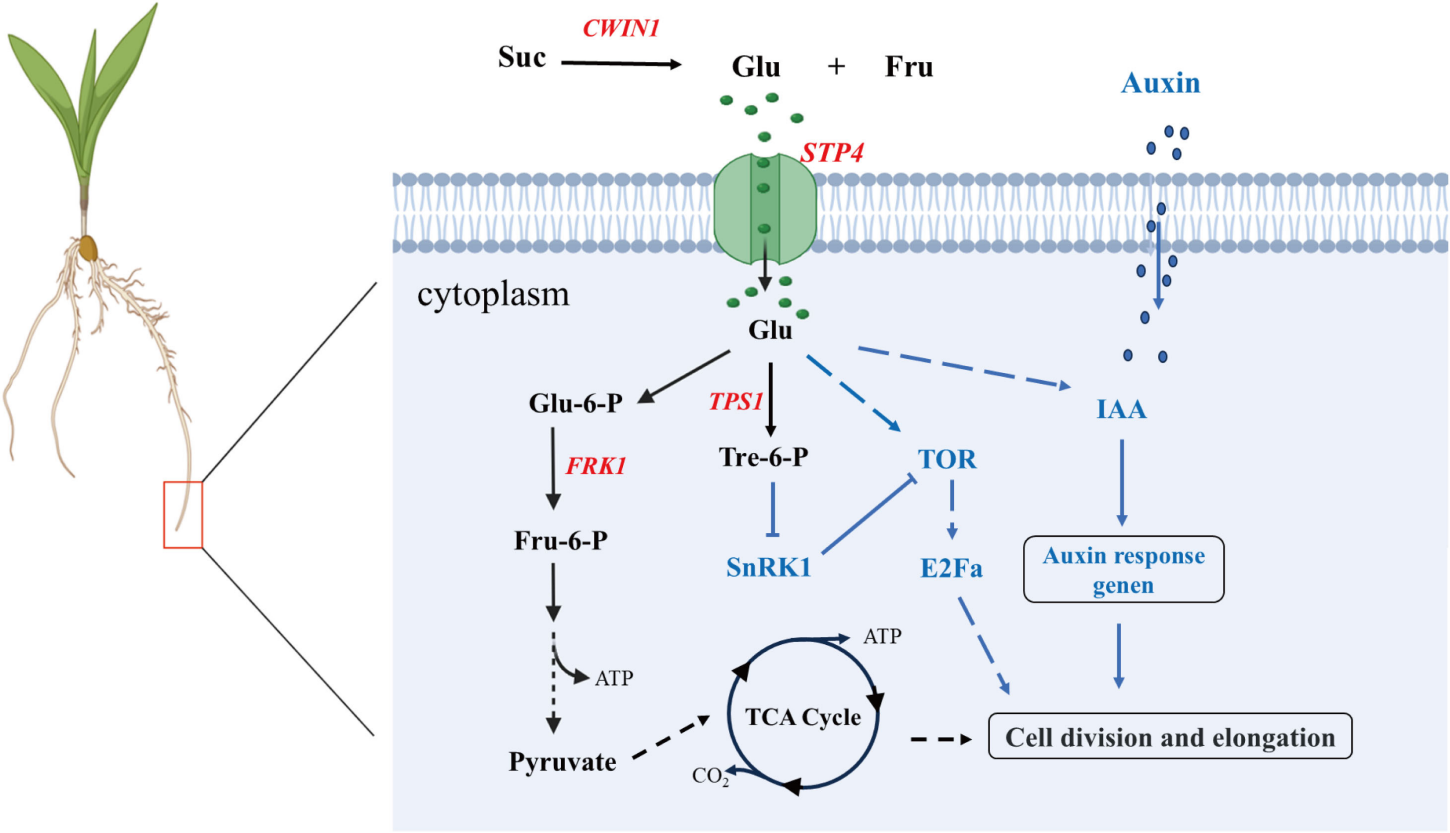
Prediction model of sugar transport protein STP4 involved in regulating maize root development. Suc: Sucrose; Glu: Glucose; Fru: Fructose; Glu-6-P: Glucose-6-Phosphate; FPK1: Fructokinase 1; Flu-6-P: Fructose-6-Phosphate; Tre-6-P: Trehalose-6-Phosphate; TOR: Target of Rapamycin; E2Fa: E2 Promoter Binding Factor a; Dashed lines indicate possible paths. Solid line with arrowhead: activation regulation; Solid line with flat head (T-bar): inhibition; Dashed line with arrowhead: Potential regulatory effects. Red: upregulated gene; Black arrows: Represent key steps in sugar metabolism; Blue arrows/dashed lines: Indicate regulatory pathways involving plant hormones and signaling molecules.

Sugar serves as an indispensable energy source for plant growth and development, and it requires the participation of sugar transporter proteins for crossing the hydrophobic barrier in plants. Transmembrane transport of sugars is a key process in adapting plant organ characteristics and overall development to plant nutritional status (Guo et al., 2023). STP4 is involved in the regulation of sucrose metabolism through the transport of monosaccharides (such as glucose and fructose), and affects the carbon allocation and energy supply of root (Ciereszko, 2018; Guo et al., 2023). During the early developmental stages of maize seedlings, energy provision primarily relies on the glucose derived from the hydrolysis of starch stored in the kernel. we found that the up-regulated expression of Zm00001d016708, encoding cell wall invertase 1 (*CWIN1*), a critical enzyme in glucose and fructose metabolism. *CWIN1* catalyzes the irreversible hydrolysis of sucrose into glucose and fructose in the apoplastic space. Subsequently, the monosaccharide transporter protein STP4 facilitates the uptake of glucose into the cytoplasm, participating the glycolysis and the tricarboxylic acid (TCA) cycle to generate ATP, thereby facilitating cell division and tissue development during early seedling growth. Additionally, the up-regulated of Zm00001d010755, which encodes trehalose-6-phosphate synthase 1 (*TPS1*), the rate-limiting enzyme for trehalose-6-phosphate (Tre-6-P) synthesis. Tre-6-P functions as a metabolic signal by directly binding to the *SnRK1* (Sucrose Non-fermenting 1-Related Kinase 1) complex, releasing its growth-suppressive effects, thereby maintaining development under fluctuating energy conditions. Furthermore, transcriptional upregulation of auxin-responsive proteins *IAA12* and *IAA13* was detected across multiple cell types (**Figure 9**). Notably, Mishra et al. demonstrated that glucose and auxin signaling interact in controlling *Arabidopsis* thaliana seedlings' root growth and development (Mishra et al., 2009). Therefore, we speculated that it may have a similar effect in maize roots. However, the molecular mechanisms underlying their functions in maize roots remain to be elucidated. Future studies should focus on characterizing these pathways to better understand their roles in maize root development.

## Conclusion

In summary, a single-cell transcriptomic atlas of maize root tips was constructed, identifying nine major cell types and revealing the cellular heterogeneity within this tissue. Although not all cell types were captured, the results offer a comprehensive view of root tip cellular diversity. Differentially expressed genes across cell subgroups were characterized, along with their associated biological pathways, underscoring key regulators of root development. Cell cycle analysis revealed distinct cyclin gene expression patterns, indicating active cell division in specific cell populations. The expression patterns of genes involved in hormone synthesis and response were revealed in maize roots, which were different from those observed in *Arabidopsis* and rice. Pseudotime analysis reconstructed the developmental trajectory from early to mature cortex cells, and WGCNA identified Zm00001d021775 (STP4) as a potential hub gene associated with root maturation and glucose metabolism. Collectively, these findings advance understanding of maize root cellular specialization and provide a valuable framework for future research into gene function, regulatory networks, and the molecular basis of root development and stress resilience.

## Data Availability

The data presented in the study are deposited in the NCBI BioProject repository, accession number PRJNA1358369.
