# Supplementary material for: Single-cell RNA sequencing reveals cellular diversity and gene expression dynamics in maize root development
Source: Front Plant Sci. 2025 Nov 27;16:1666531. doi: 10.3389/fpls.2025.1666531 (PMC12695853; doi:10.3389/fpls.2025.1666531)
Supplement: Supplementary file 1 [file DataSheet1.zip › Supplementary_Material/Supplementary_Material.docx]

Supplementary Material


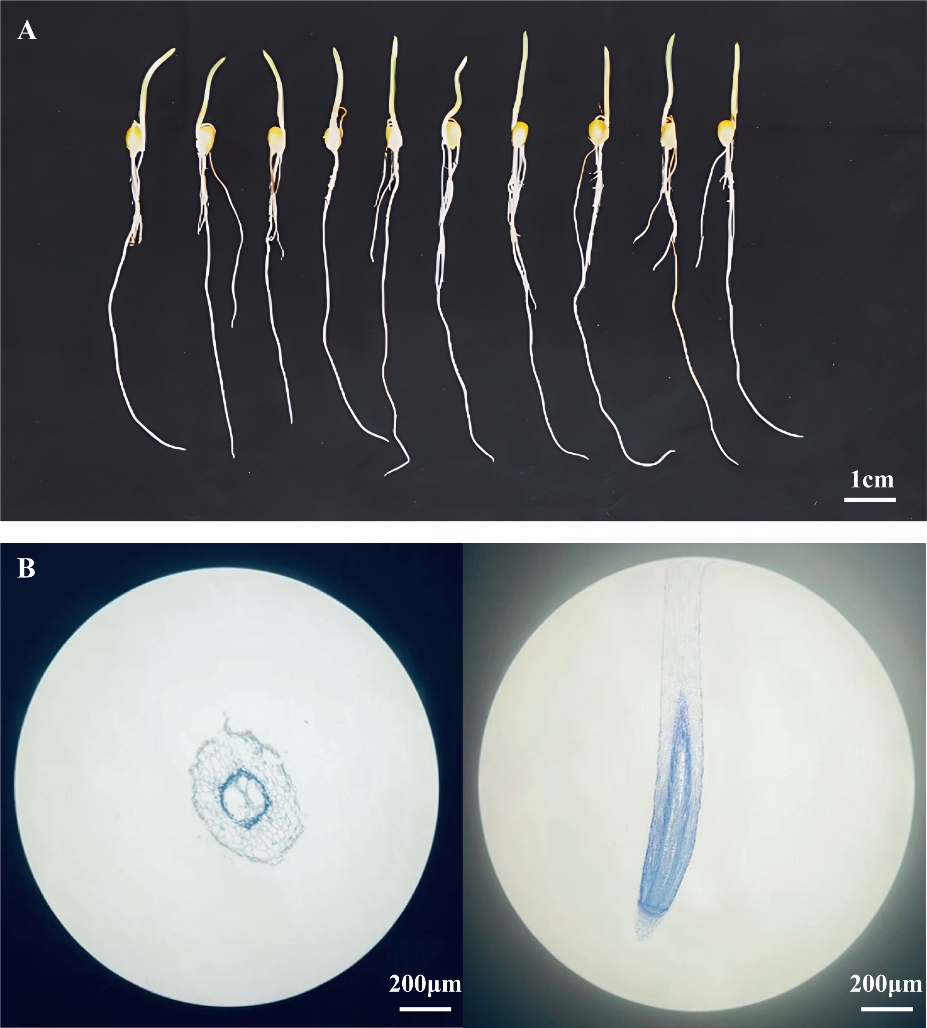


**Supplementary Figure 1.** Phenotype of seedling used for performing scRNA-seq. (**A**) Phenotype of 7-day-old maize seedlings. (**B**) Paraffin section of maize root tip.

**
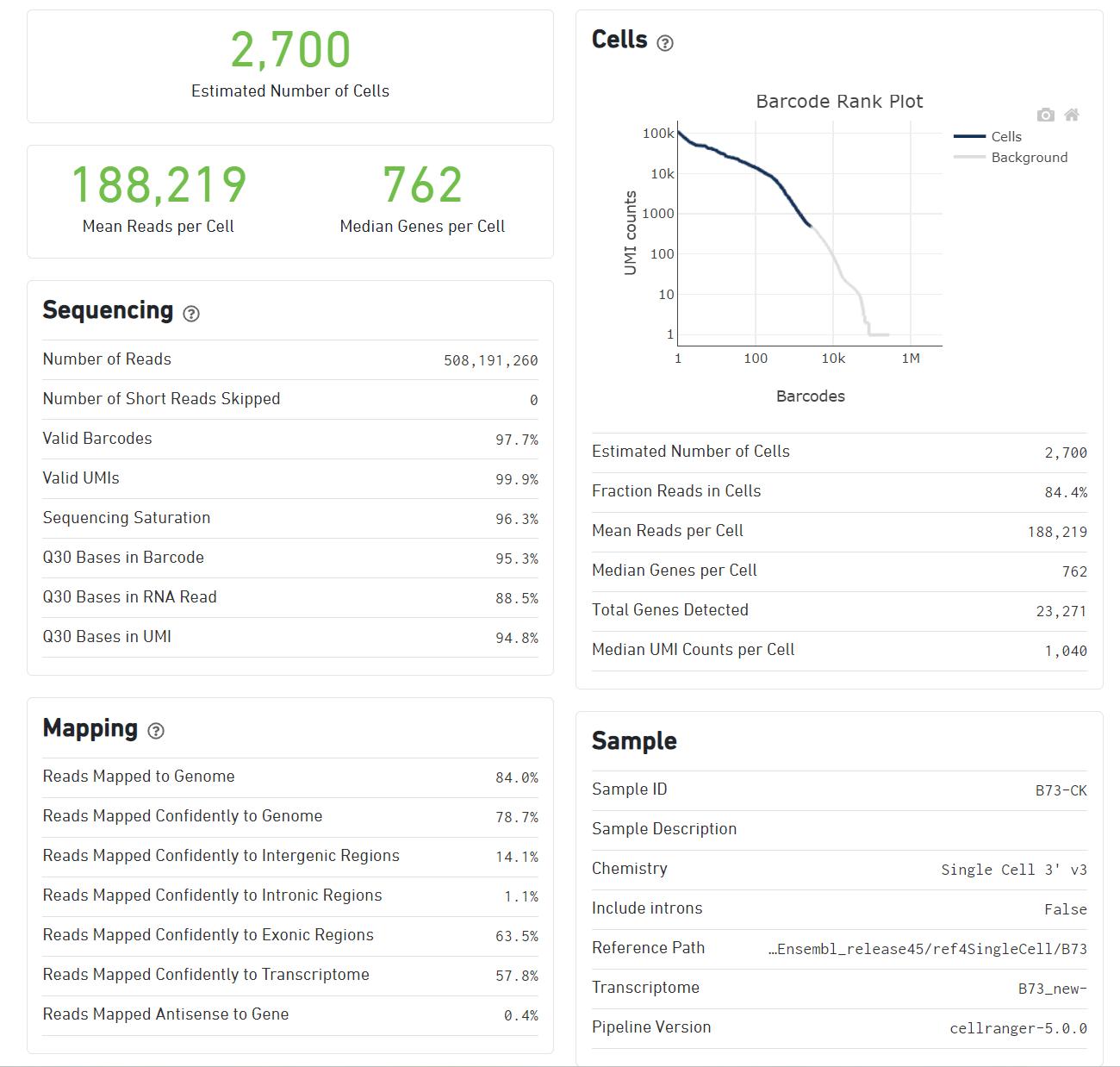
**

**Supplementary Figure 2.** Brief chart of Cell Ranger software report.


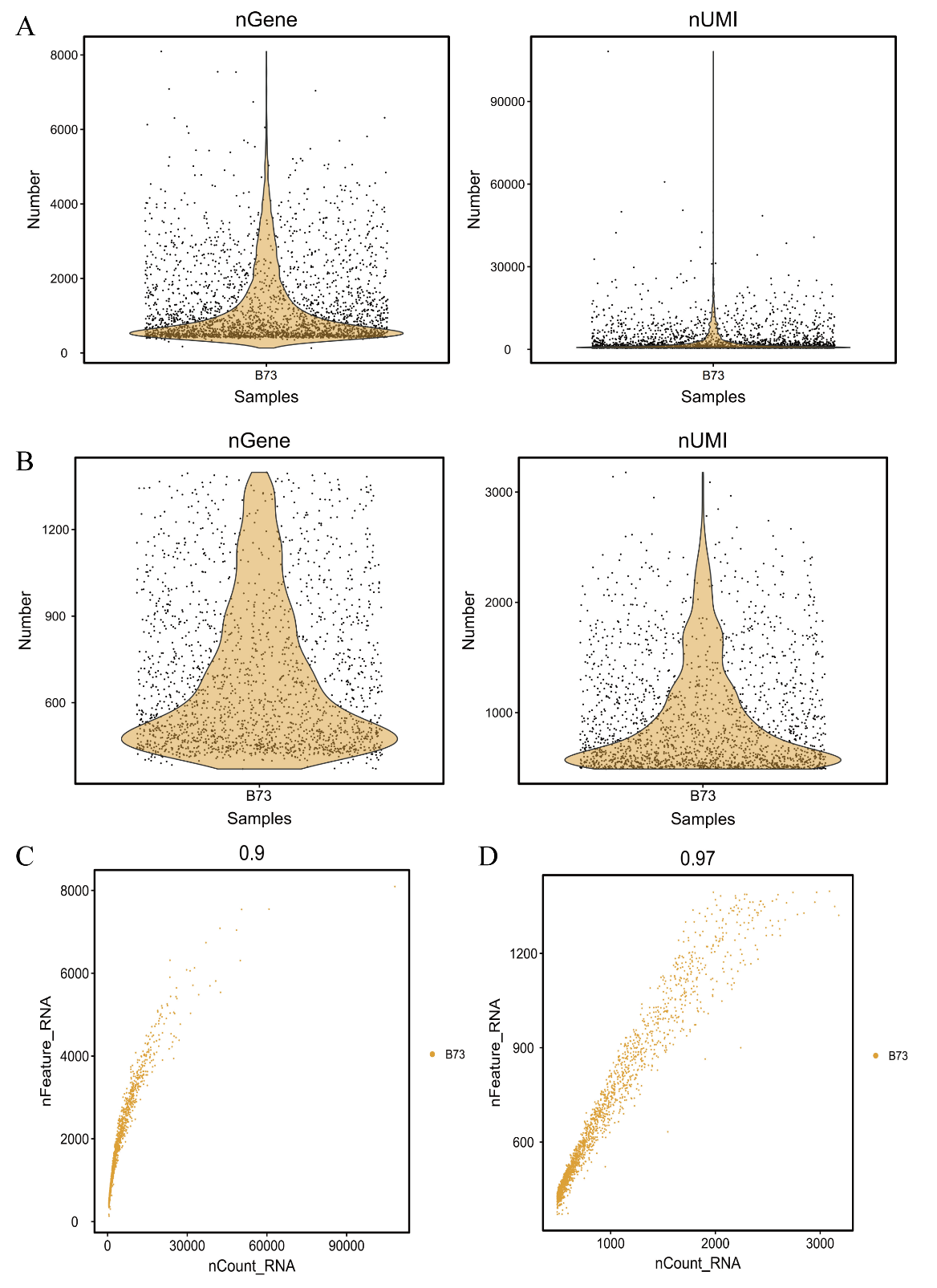


**Supplementary Figure 3.** Data quality control determined the medium number of gene and UMI. (**A**, **B**) Distribution charts of the basic information of cells in nUMI and nGene before (A) and after filtration (B). (**C**, **D**) Correlation coefficient between the UMI and gene before filter (C) and after filter (D).

**
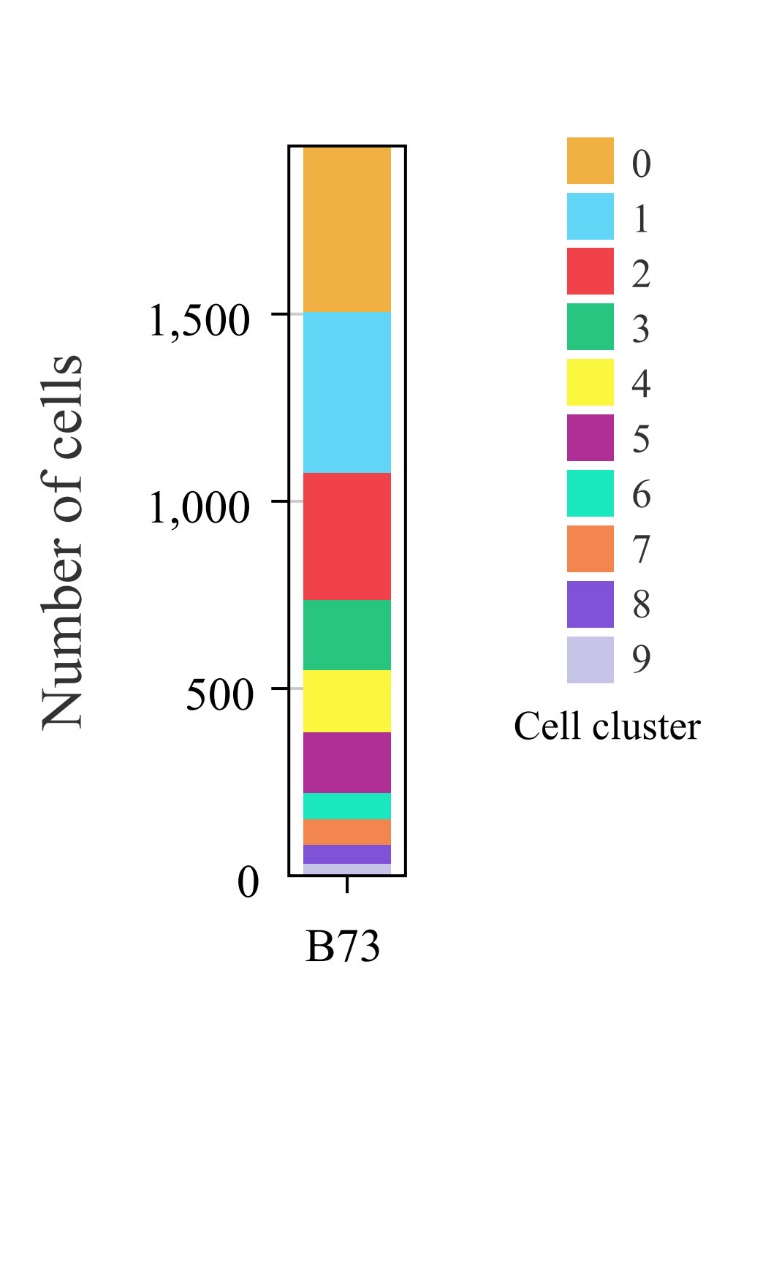
**

**Supplementary Figure 4.** The number of cells in each maize root cell cluster.

**
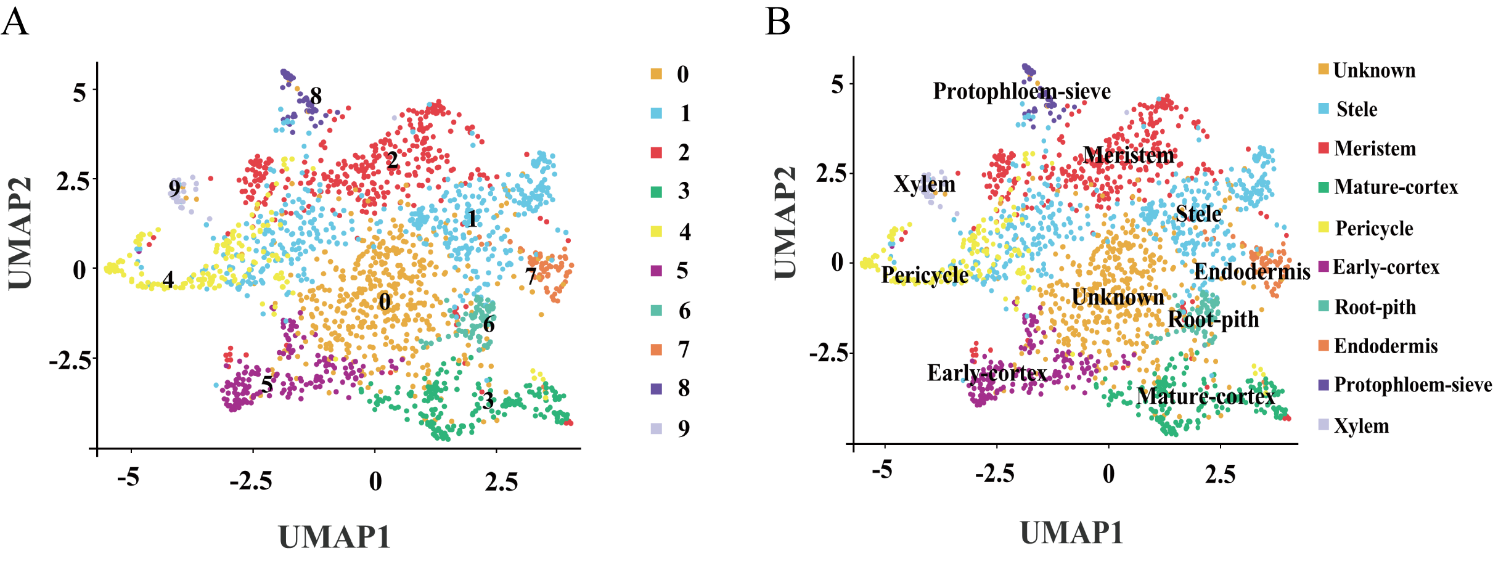
**

**Supplementary Figure 5.** UMAP visualization of the 10 cell clusters. (**A**, **B**) UMAP visualization for the identification of 10 cell clusters in root tips. Each dot indicates a single cell. Colors in the diagram of the root tip indicate corresponding cell clusters.


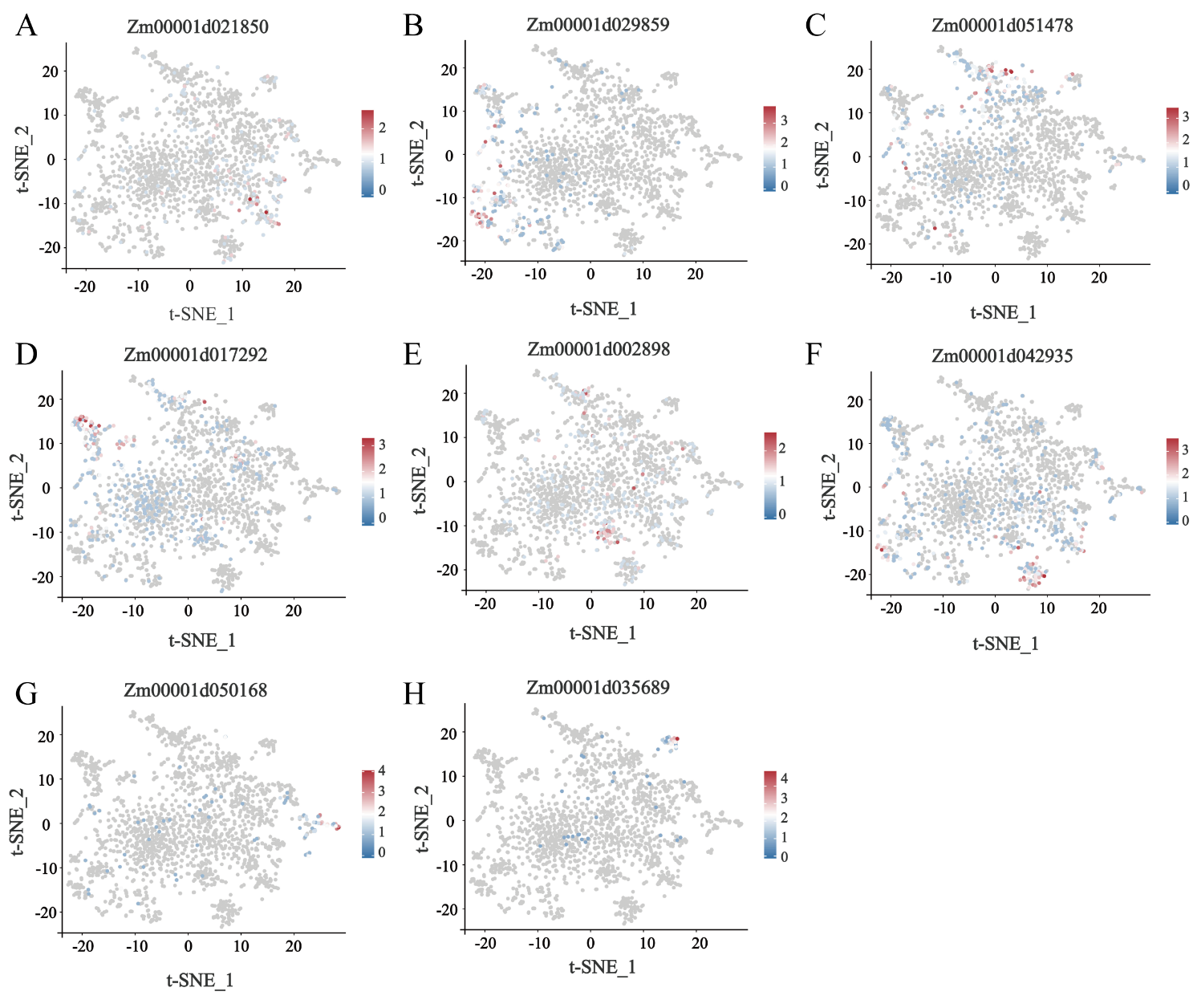


**Supplementary Figure 6.** t-SNE visualization of expression of representative cell type marker genes. (**A-H**) The expression of representative cell type marker genes distributed in tSNE map. Each dot indicates a single cell. The color on the dots indicates the expression level.


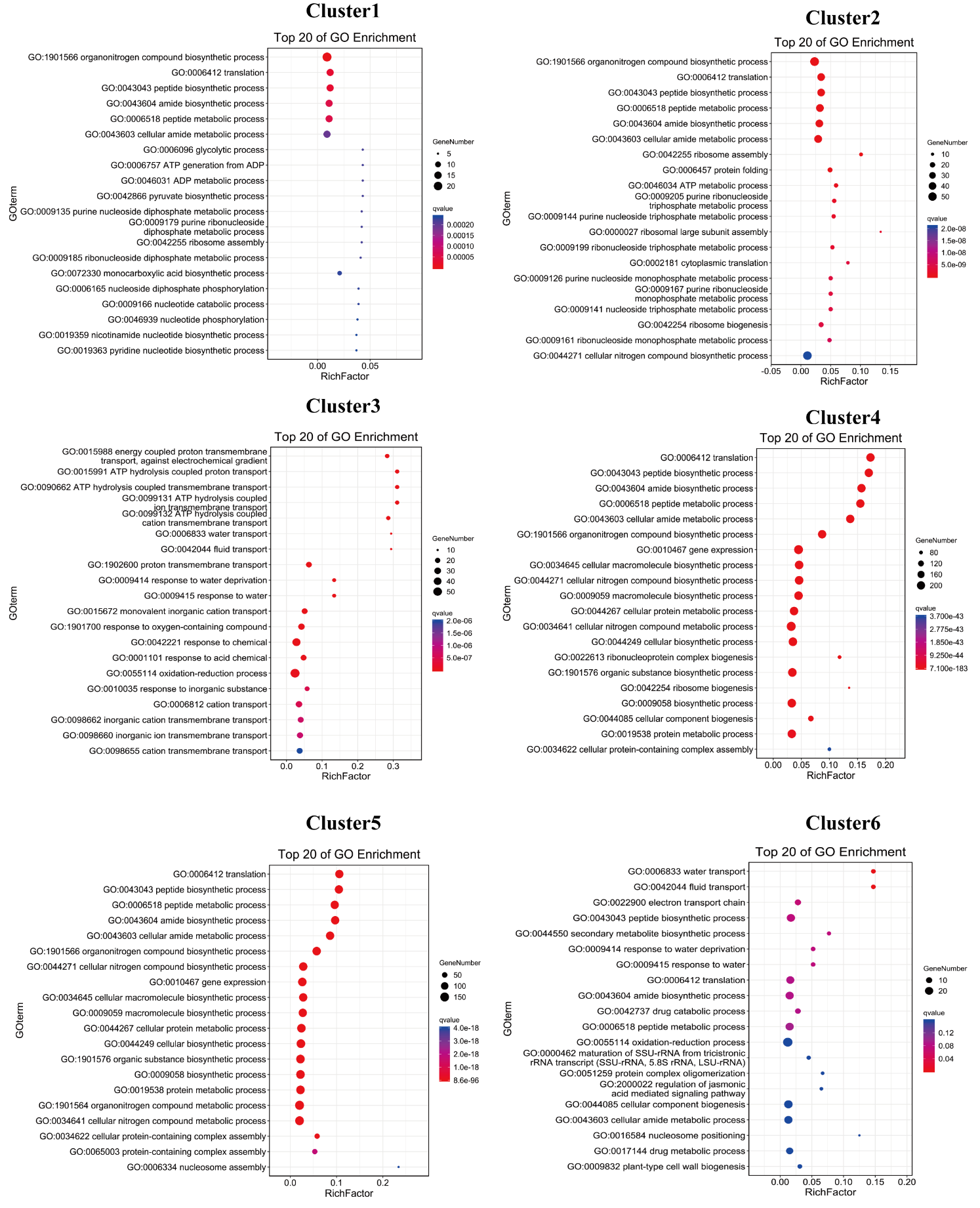


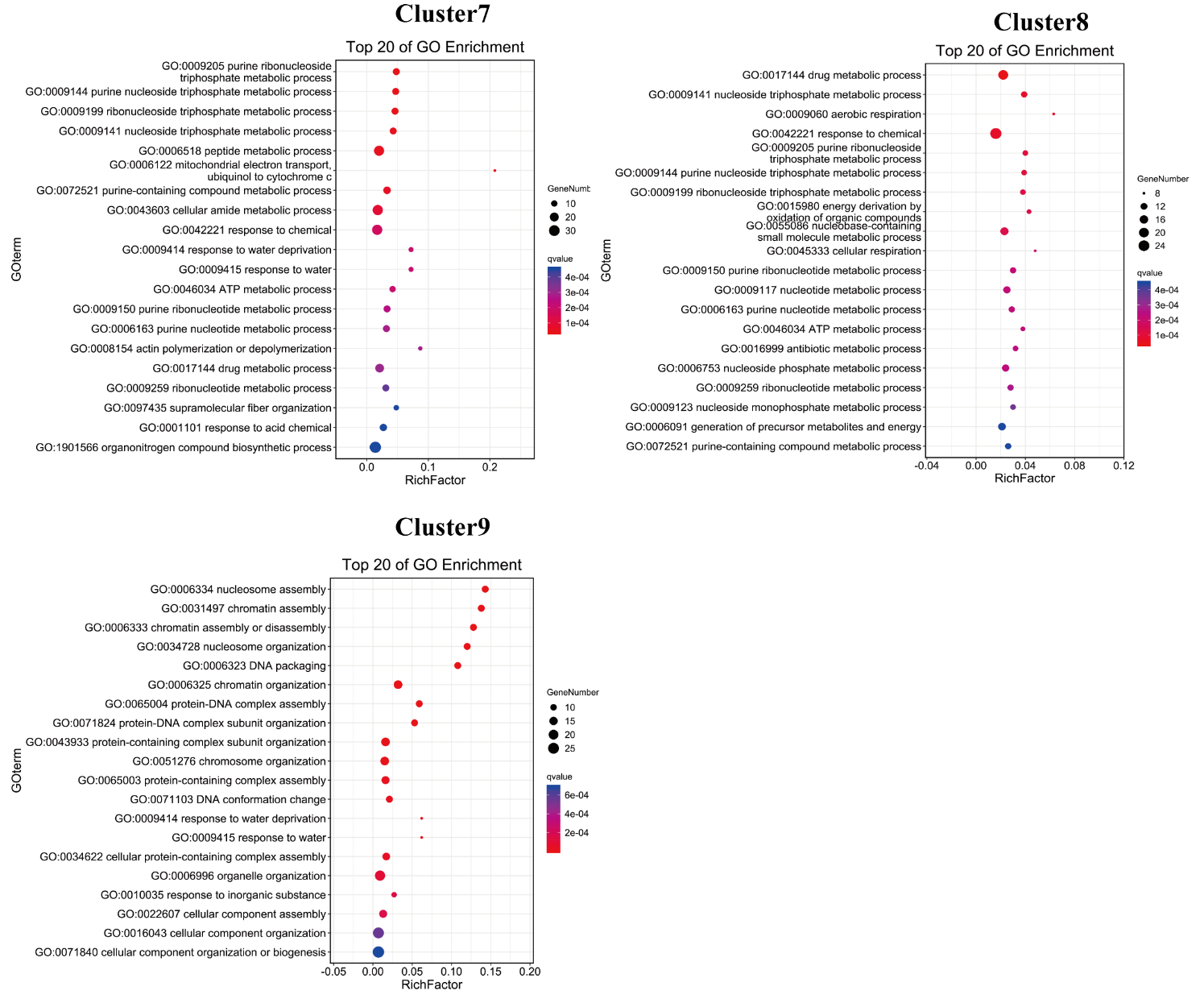


**Supplementary Figure 7.** GO enrichment analysis of up-regulated genes of each cell cluster in maize root tip.


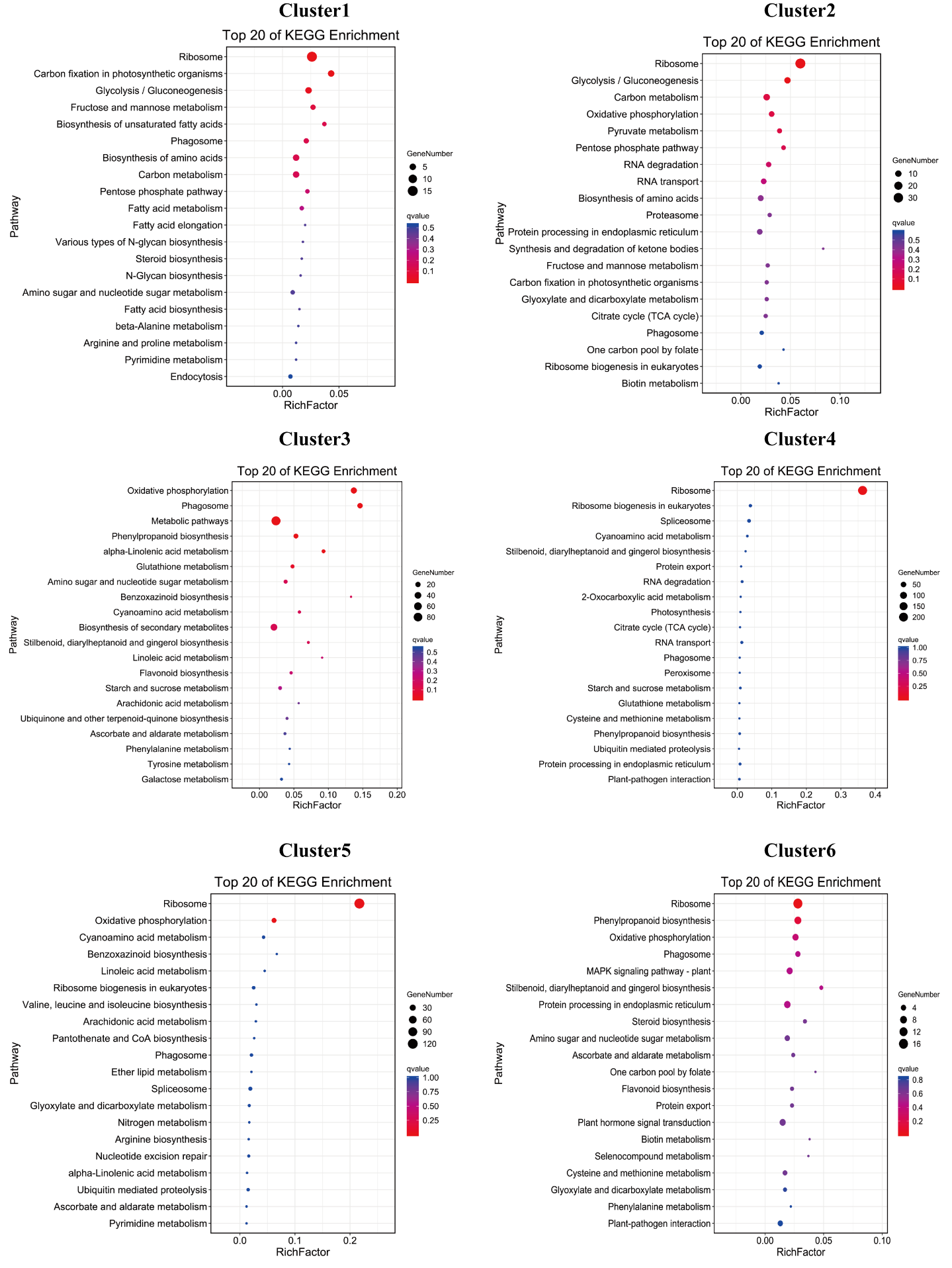


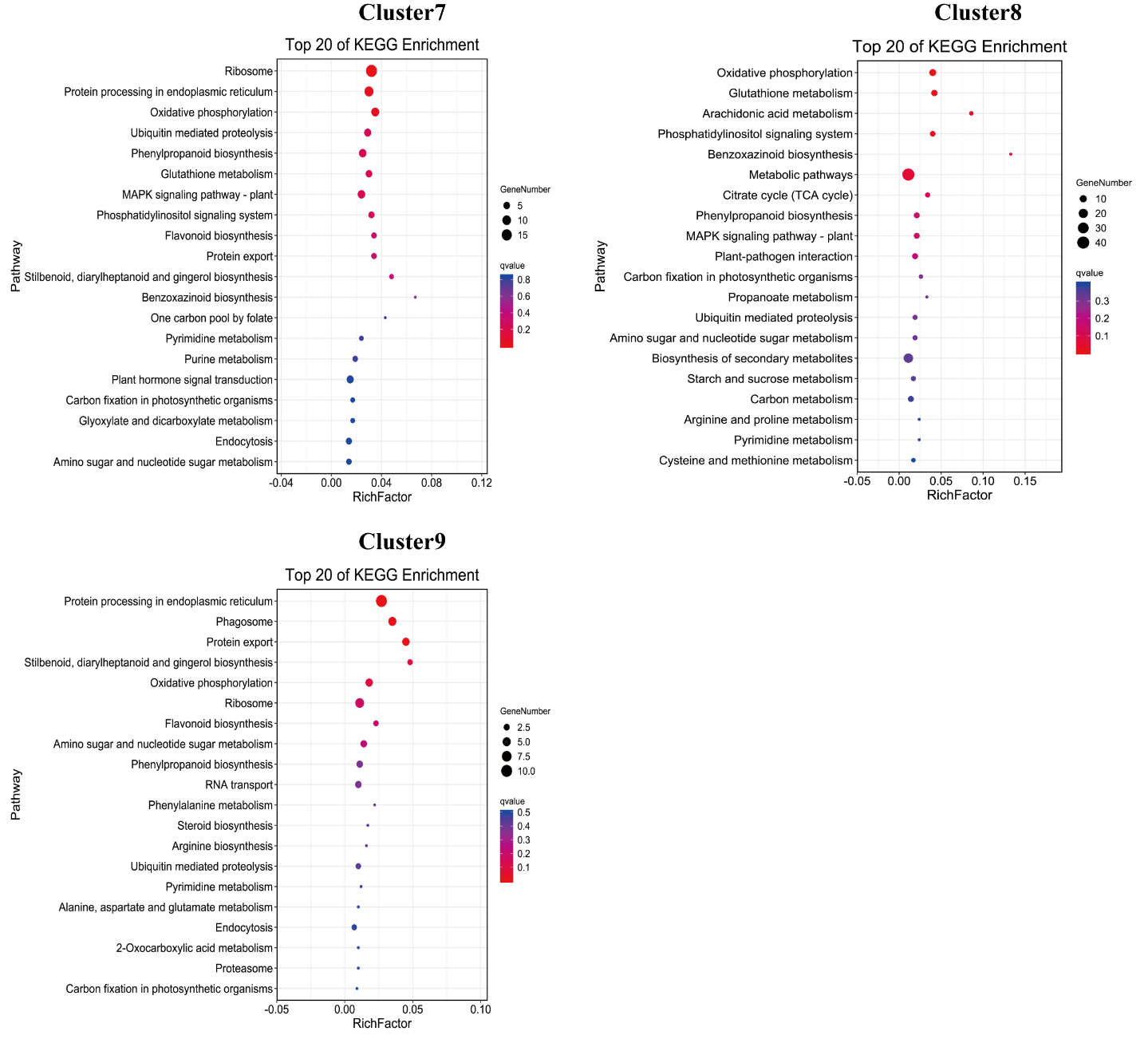


**Supplementary Figure 8.** KEGG enrichment analysis of up-regulated genes of each cell cluster in maize root tip.


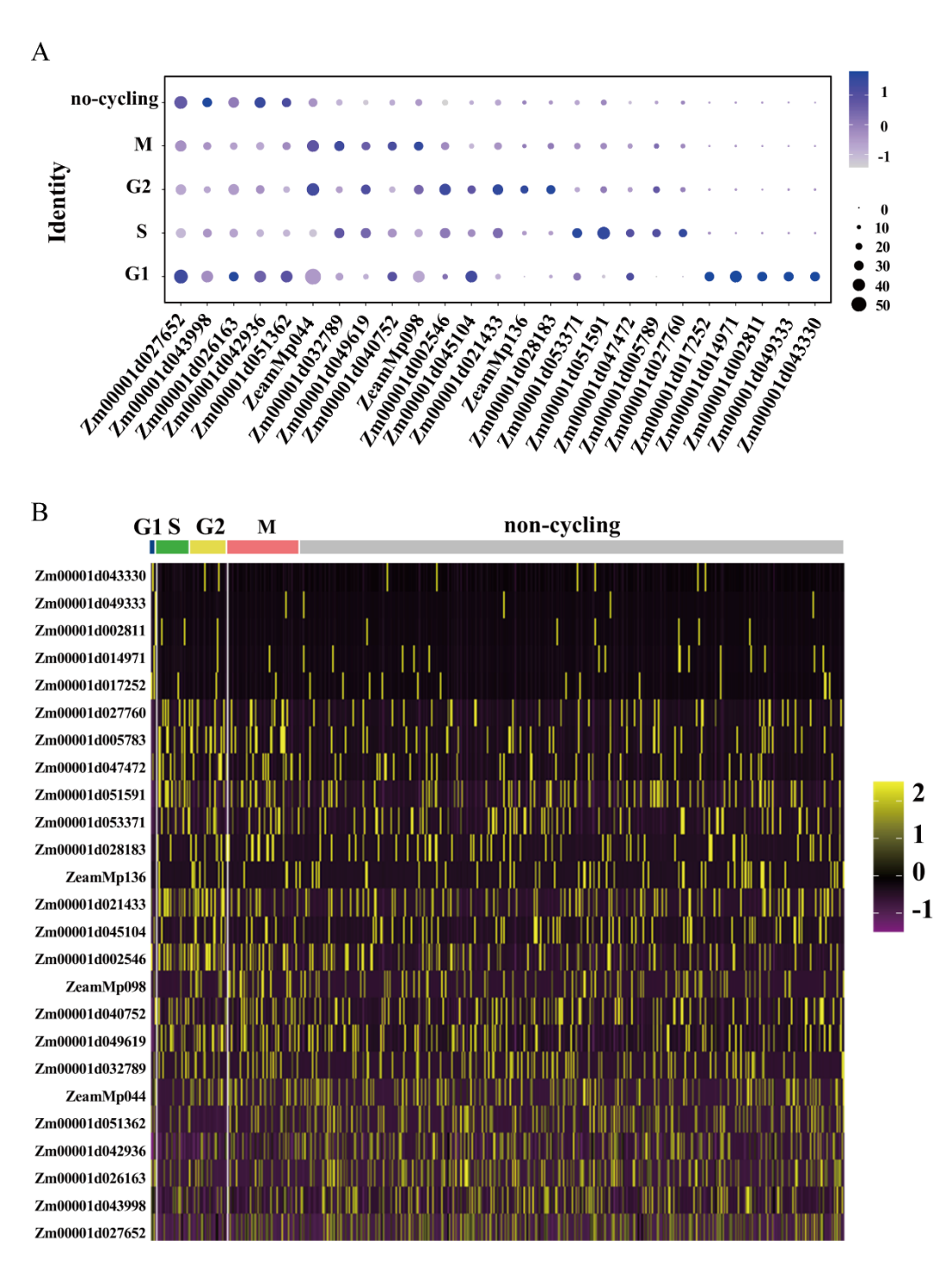


**Supplementary Figure 9.** Cell cycle analysis of 10 cell clusters in maize root tip. (**A**) Bubble plot presenting the expression levels of cyclin gene in different cell cycles. (**B**) Heatmap presenting the expression levels of cyclin gene in different cell cycles.


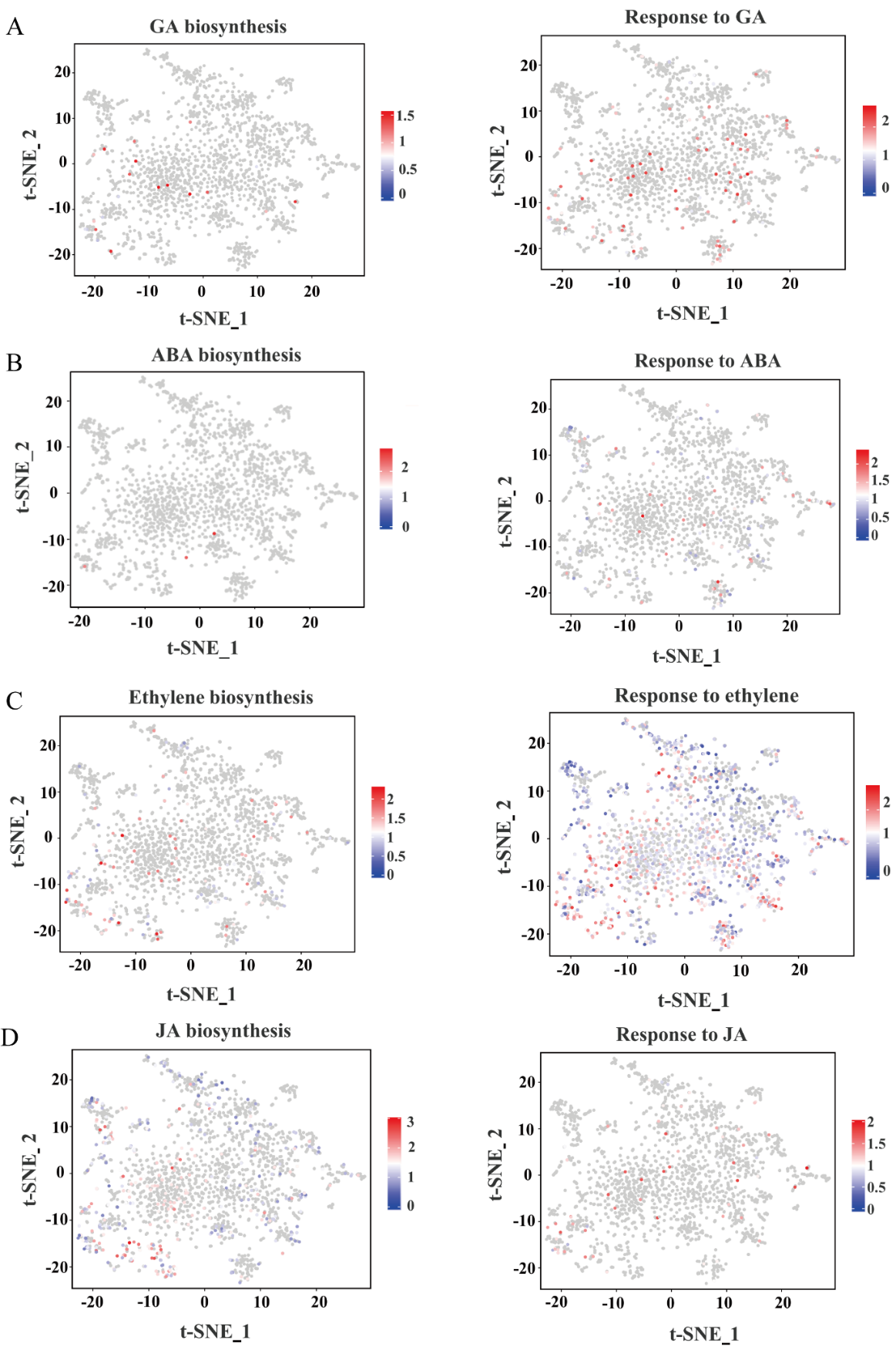


**Supplementary Figure 10.** t-SNE visualization of expression patterns of related to GA (**A**), ABA (**B**), Ethylene (**C**), and JA biosynthesis and response gene (**D**). The colors represent expression levels of these genes in individual cells.


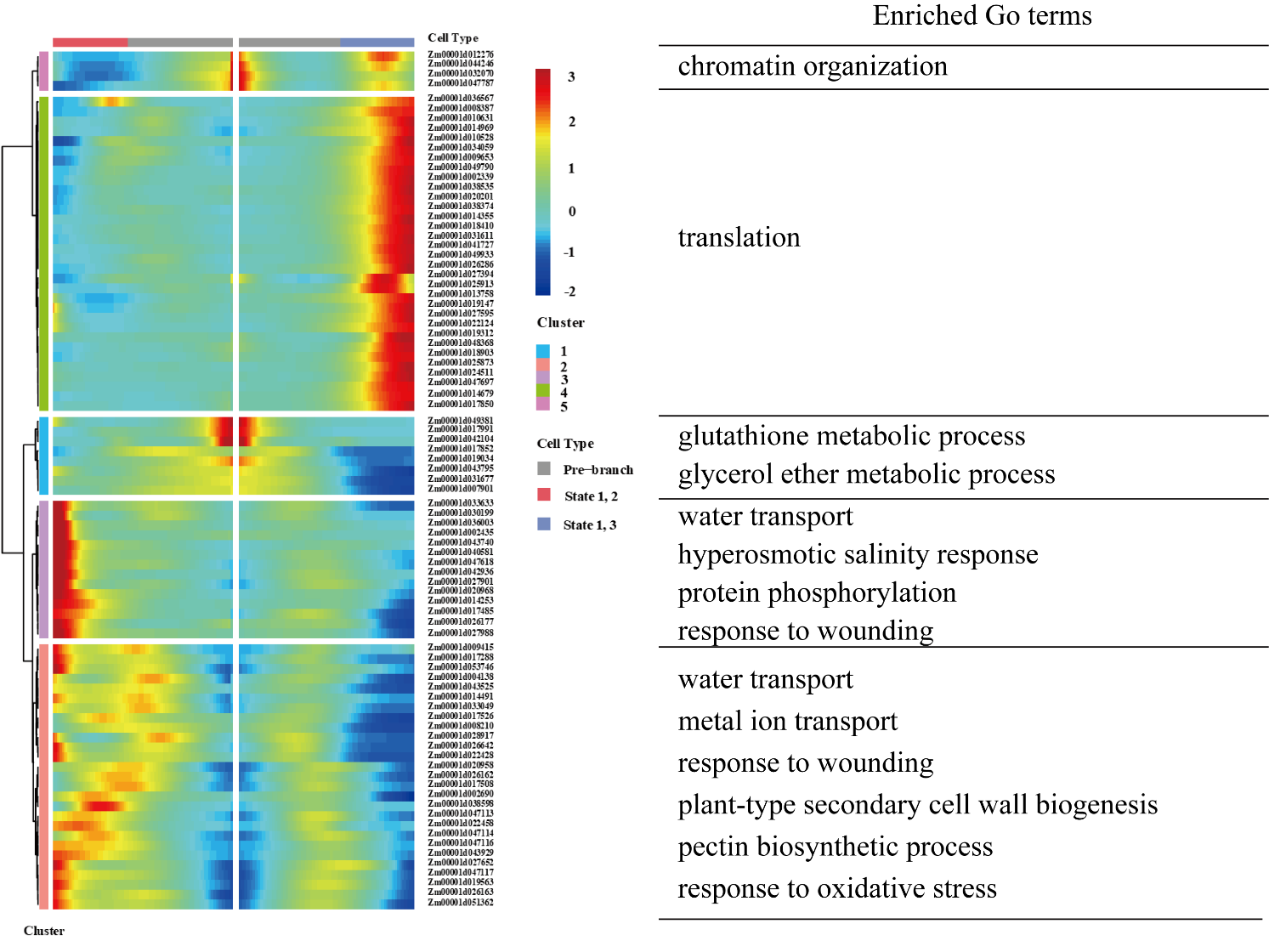


**Supplementary Figure 11.** Heatmap of cell differentiation fate DEGs. Each row represents one gene. Representative GO terms for each cluster are shown on the right.
